# Prenatal Amino Acid Supplementation to Improve Fetal Growth: A Systematic Review and Meta-Analysis

**DOI:** 10.3390/nu12092535

**Published:** 2020-08-21

**Authors:** Fieke Terstappen, Angela J. C. Tol, Hendrik Gremmels, Kimberley E. Wever, Nina D. Paauw, Jaap A. Joles, Eline M. van der Beek, A. Titia Lely

**Affiliations:** 1 Department for Developmental Origins of Disease (DDOD), Wilhelmina Children’s Hospital University Medical Center Utrecht, 3584 CX Utrecht, The Netherlands; 2 Department of Obstetrics, Wilhelmina Children’s Hospital, University Medical Center Utrecht, 3584 CX Utrecht, The Netherlands; N.D.Paauw-2@umcutrecht.nl (N.D.P.); A.T.Lely@umcutrecht.nl (A.T.L.); 3 Department of Pediatrics, University Medical Center Groningen, 9700 RB Groningen, The Netherlands; a.j.c.tol@umcg.nl (A.J.C.T.); Eline.VANDERBEEK@danone.com (E.M.v.d.B.); 4 Department of Nephrology and Hypertension, University Medical Center Utrecht, 3584 CX Utrecht, The Netherlands; H.Gremmels@umcutrecht.nl (H.G.); J.A.Joles@umcutrecht.nl (J.A.J.); 5 Systematic Review Center for Laboratory Animal Experimentation (SYRCLE), Department for Health Evidence, Radboud Institute for Health Sciences, Radboud University Medical Center, 6525 GA Nijmegen, The Netherlands; Kim.Wever@radboudumc.nl; 6 Danone Nutricia Research, 3584 CT Utrecht, The Netherlands

**Keywords:** amino acids, arginine, birth weight, branched chain amino acid, fetal growth restriction, meta-analysis, methyl donor, pregnancy

## Abstract

Aberrant fetal growth remains a leading cause of perinatal morbidity and mortality and is associated with a risk of developing non-communicable diseases later in life. We performed a systematic review and meta-analysis combining human and animal studies to assess whether prenatal amino acid (AA) supplementation could be a promising approach to promote healthy fetal growth. PubMed, Embase, and Cochrane libraries were searched to identify studies orally supplementing the following AA groups during gestation: (1) arginine family, (2) branched chain (BCAA), and (3) methyl donors. The primary outcome was fetal/birth weight. Twenty-two human and 89 animal studies were included in the systematic review. The arginine family and, especially, arginine itself were studied the most. Our meta-analysis showed beneficial effects of arginine and (*N*-Carbamyl) glutamate (NCG) but not aspartic acid and citrulline on fetal/birth weight. However, no effects were reported when an isonitrogenous control diet was included. BCAA and methyl donor supplementation did not affect fetal/birth weight. Arginine family supplementation, in particular arginine and NCG, improves fetal growth in complicated pregnancies. BCAA and methyl donor supplementation do not seem to be as promising in targeting fetal growth. Well-controlled research in complicated pregnancies is needed before ruling out AA supplements or preferring arginine above other AAs.

## 1. Introduction

Divergence in fetal growth—both under- and overgrowth—remains a leading cause of perinatal mortality and morbidity [1,2]. Fetal growth divergence has been associated with the development of non-communicable diseases later in life, including cardio-metabolic disorders [3,4,5,6,7,8].

Normal fetal growth requires adequate amino acid (AA) supply during all trimesters, which depends on the placental capacity to transfer AAs from the maternal to fetal side [9]. Several factors influence this transfer capacity, such as maternal plasma AA concentrations, utero-placental blood flow, and placental surface area [10].

A disruption in fetal supply of AAs might contribute to fetal under- or overgrowth. The major cause of fetal growth restriction (FGR) is placental insufficiency, which often co-occurs with hypertensive disorder during pregnancy. Decreased utero-placental blood flow could result in reduced placental transfer of AAs and consequently FGR. Lower circulating levels of AAs of the arginine family and branched chain AAs (BCAA) and reduced expression or activity of placental AA transporters are indeed observed in FGR pregnancies [11,12,13,14,15,16]. On the other hand, fetal overgrowth is observed in gestational diabetes mellitus (GDM) in which increased circulating AA levels interact with insulin sensitivity, and increased maternal glucose stimulates nutrient transport over the placenta [17,18,19,20].

Oral supplementation of AAs during pregnancy could be an effective—and relatively safe—therapeutic or prophylactic solution to improving perinatal and long-term health. The arginine family, BCAAs, and methyl donors form three interesting supplementation groups by virtue of their influence on fetal growth. The arginine family plays a key role in placental growth and development through nitric oxide (NO) and polyamine syntheses and through the mammalian target of rapamycin (mTOR) pathway [9,12,21,22,23]. Arginine also stimulates creatine production and skeletal muscle protein synthesis [24]. BCAAs possess strong insulinogenic and anabolic effects, and these essential AAs mediate lean body mass growth through mTOR [12,25]. Methyl donors stimulate fatty acid catabolism [26]. Their ability to donate methyl groups facilitates genetic and epigenetic regulation of placental and fetal programming [27]. The effect of AA supplementation during pregnancy has been studied in both humans and animals, but a clear overview of the resulting effects is currently lacking.

This systematic review and meta-analysis evaluates the effect of oral supplementation of the three AA groups on fetal growth in complicated and normal-growth pregnancies. Considering the altered circulating levels of the specific AAs, the activity of placental transporters, and the hypothesized mechanism of action, we speculate that AAs from the arginine family or BCAAs normalize fetal undergrowth, while methyl donors normalize fetal overgrowth. By including different AAs in animal and human studies in one meta-analysis, we aim to identify the most effective AA (group) and other modifiable factors (e.g., dose). This will contribute towards future study designs aimed at developing an AA-based supplementation strategy to prevent fetal growth divergence and its sequels.

## 2. Materials and Methods

### 2.1. Study Protocol

This systematic review was conducted according to a prespecified protocol registered at PROSPERO for animal studies (CRD42018098779; based on [28]) and human studies (CRD42018095995) (https://www.crd.york.ac.uk/PROSPERO). The sparse amendments to the review protocol that were made post hoc are reported in the Appendix. This review is reported according to the Preferred Reporting Items for Systematic Reviews and Meta-Analyses guidelines [29]. No language or publication date restrictions were applied.

### 2.2. Data Sources and Search Strategy

On 25th July 2018, we searched Pubmed, Embase (via OVID), and the Cochrane Library database to identify animal and human studies reporting on prenatal supplementation of 14 AAs falling within the following three groups: (1) arginine family: arginine, citrulline, glutamate, glutamine, asparagine, aspartate, proline, and ornithine; (2) BCAA: leucine, iso-leucine, and valine; and (3) methyl donors: cysteine, methionine, and the AA derivate choline. Search strings are provided (Table A1, Table A2 and Table A3).

### 2.3. Study Selection: Inclusion and Exclusion Criteria

Two independent investigators (F.T. and A.T.) screened articles for inclusion using predefined inclusion and exclusion criteria in Early Review Organizing Software (EROS, version 2.0)), as described in detail in the Appendix. To be eligible for inclusion, studies needed to (1) to be performed in mammals with a normal-growth pregnancy or a complicated pregnancy resulting in fetal growth divergence: FGR, pre-eclampsia (PE), pregnancy-induced hypertension (PIH), GDM/diabetes mellitus, or prematurity; (2) to study the effects of oral supplementation with one of the 14 AAs for more than one day (at lib) or at least twice (as bolus) during pregnancy; and (3) to report one of the following outcomes: fetal/birth weight; maternal blood pressure (BP); maternal glucose or insulin levels; gestational weight gain; or development of pregnancy complications in human risk population including FGR, PE, PIH, GDM, prematurity, and neonates born small or large for gestational age (SGA or LGA). For human studies, we only included randomized controlled trials. The complete list of exclusion criteria is reported in the Appendix.

### 2.4. Data Extraction

Data were extracted in duplicate by F.T. and A.T. We extracted data on study characteristics, such as supplementation strategy and the (gestational) day of measurement, and for each outcome mean, SD, and number of subjects per experimental group were noted (see Appendix for more details).

### 2.5. Assessment of Risk of Bias and Study Quality

Risk of bias was assessed in duplicate by F.T. and A.T. using the risk of bias tools from SYRCLE for animal studies [30] and from the Cochrane Collaboration (Review Manager 5.3.5, The Nordic Cochrane Centre, Copenhagen, Denmark) for human controlled trials. Adjustments to the tools are described in Appendix. Studies were not excluded based on poor study quality.

### 2.6. Meta-Analysis

Meta-analyses were performed separately for normal-growth versus complicated pregnancies versus pregnancies at-risk of complications. All animal species were pooled together, and the analyses were performed for each AA group for each outcome only when more than five studies could be included. Fetal/birth weight (primary outcome) was compared between groups as a ratio of means (ROM) and, in humans, additionally presented as a mean difference (MD). Maternal BP, blood glucose or insulin levels, and gestational weight gain were presented as an MD, and development of pregnancy complication was presented as an odds ratio (OR). Pooled effect size estimates are presented with their 95% confidence intervals (95% CI). Data were analyzed using random- or mixed effects models, using nesting if multiple cohorts from one study were included [31].

Meta-regression analyses were performed to study effects of modifiable factors in the complicated pregnancy group only, and were only performed if at least two studies per category were present. When studies reported data on multiple cohorts (e.g., multiple dose), then these were included in the meta-analysis as independent comparisons. Meta-regression was performed on species, type of pregnancy complications, administration timing (full pregnancy vs. partly), and scheme (continuous vs. interval), intervention type (prevention or treatment), and control diet (isonitrogenous vs. not isonitrogenous in arginine family). For BP analysis, we used mean arterial pressure and, when not available, systolic BP. For the dose–response curves, a metabolic weight conversion was applied by a linear scaling exponent of 0.75 to correct for interspecies pharmacokinetic conversion [31].

A two-sided *p*-value below 0.05 was considered significant. Potential publication bias was visually examined in funnel plots and tested by Egger’s regression when over twenty studies reported an outcome. Heterogeneity (I^2^) among studies > 50% was considered significant. An influential case analysis was performed by examining residuals, weights, and Cook’s distances of model fits. A sensitivity analysis was executed by removing influential cases and by shifting cut-outs for meta-regression of MD or ROM. R software (v. 3.5.3, The R Core team, Auckland, New Zealand) and the Metafor package were used for all statistical analyses [32].

## 3. Results

### 3.1. Study Selection and Overall Study Characteristics

**Figure A1:**
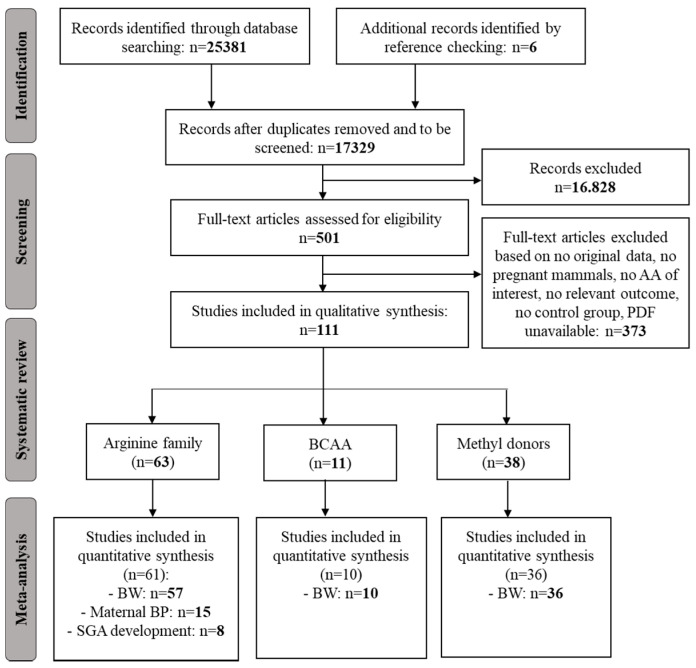
Flow chart of the study selection process: Our search strategy retrieved 17,329 unique hits, of which we included 111 studies reporting on amino acid (AA) supplementation in our systematic review. Of these, 63 studies reported on arginine supplementation, 11 reported on branched-chain amino acids (BCAA) supplementation, and 38 reported on methyl donor supplementation. We pooled data on the effect of arginine supplementation on birth weight (BW) in 57 studies, on maternal blood pressure (BP) in 15 studies, and on small for gestational (SGA) development in risk populations in 8 studies. We were able to pool data on the effect of BCAA on BW in 10 studies and of methyl donor supplementation in 36 studies. Adapted from PRISMA [29].

The search resulted in 17.329 hits. The majority of exclusions during the full-text screen was based on no in vivo studies on pregnant animals or humans followed by no supplementation of the amino acids of interest and resulted in 501 studies for full-text screening, of which we included 111 studies in our systematic review (Figure A1). We included 5 mouse, 40 rat, 4 guinea pig, 1 rabbit, 9 sheep, 23 pig, 7 cow, and 22 human studies. None of the included studies reported on asparagine or ornithine supplementation. Table A4 summarizes which outcome was reported per study and total data-extraction per outcome is reported in Table A5, Table A6, Table A7, Table A8 and Table A9.

### 3.2. Overview Performed Meta-Analyses

We performed meta-analyses on fetal/birth weight following supplementation with AA in the arginine family, BCAA, and methyl donors. Regarding maternal BP and development of SGA, the arginine family was the only AA group for which a meta-analysis could be performed. A meta-analysis on the development of other pregnancy complications was not possible. Data for gestational weight gain were not pooled because, (1) without individual participant data, we had to estimate the mean weight gain and SD for studies that did report gestational weight at two different time points during pregnancy per group; (2) studies reported gestational weight gain over different gestational time periods, which did not consistently match the supplementation periods; and (3) gestational periods are very different between species. Too few studies reported on glucose levels to pool these data. No data on insulin resistance (HOMA-IR) were found. Results on glucose and gestational weight gain are described in the Appendix.

### 3.3. Arginine Family

#### 3.3.1. Effect of Prenatal AA in Arginine Family on Fetal Growth

##### Study Characteristics

Data were extracted on fetal growth in response to prenatal supplementation of arginine family AA from 47 animal studies (1 mouse [33], 18 rat [34,35,36,37,38,39,40,41,42,43,44,45,46,47,48,49,50,51], 6 sheep [52,53,54,55,56,57], 20 pig [58,59,60,61,62,63,64,65,66,67,68,69,70,71,72,73,74,75,76,77], and 12 human studies [78,79,80,81,82,83,84,85,86,87,88,89] (Table A5). Most studies were supplemented with arginine (*n* = 47) [33,34,35,36,37,44,45,46,47,48,49,50,51,52,53,54,55,56,57,58,59,60,61,62,63,64,69,70,71,72,73,74,75,76,77,78,79,80,81,82,83,84,85,86,87,88,89], followed by glutamate (*n* = 10) [40,41,54,55,56,57,63,65,66,77], citrulline (*n* = 3) [38,39,44], glutamine (*n* = 2) [42,67], proline (*n* = 1) [68], and aspartic acid (*n* = 1) [43]; 7 studies had two treatments arms, 6 studies with arginine and (*N*-Carbamyl) glutamate (NCG) [54,55,56,57,63,77] and 1 study with arginine and citrulline [44]. The human studies, all supplementing arginine, were performed in Poland (*n* = 4) [84,85,86,87], Italy (*n* = 2) [79,83], Norway (*n* = 1) [89], France (*n* = 1) [80], Mexico (*n* = 1) [78], USA (*n* = 1) [82], China (*n* = 1) [81], and India (*n* = 1) [88].

##### Meta-Analyses

**Figure 1:**
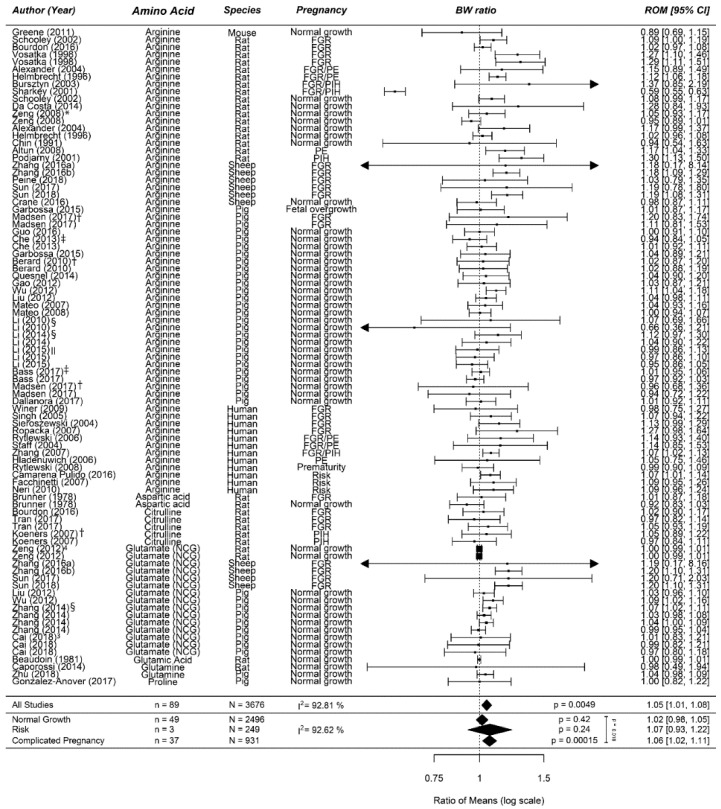
Meta-analysis on prenatal supplementation of amino acids from the arginine family on fetal/birth weight (BW): While there was no effect of prenatal supplementation of amino acids from the arginine family in normal-growth pregnancies, it increased the birth weight ratio in a risk population and in complicated pregnancies. The data is ordered within each amino acid from smallest to largest animal. Data represent pooled estimates expressed as a ratio of means (ROM) with a 95% confidence interval (CI) using a random effect model. Residual I^2^ is shown. Some studies had multiple cohorts and are distinguishable in this figure by the following: * supplementation during full pregnancy in this upper line compared to partial in the next line; † this upper line is female offspring compared to the next line which is male offspring; ‡ in this upper line, the supplementation period was shorter compared to the next line(s); § in this upper line, the daily dose is lower compared to the next line(s); || in this upper line, primigravid animals were used compared to the next two lines of multigravida animals; and in the last two lines, the dose differed with the first one being the highest dose. FGR, fetal growth restriction; I^2^, heterogeneity; NCG, *N*-(Carbamyl) glutamate; PE, preeclampsia; PIH, pregnancy-induced hypertension.

Supplementation of prenatal AA from the arginine family increases birth weight by 6% (1.06 (1.02; 1.11)) in complicated pregnancies (Figure 1). No effect was observed in normal-growth pregnancies (1.01 (0.98; 1.05)) or the risk population (1.07 (0.93; 1.22)). In animal studies only, no differences were observed in normal pregnancies and arginine increased birth weight by 8% (1.08 (1.03; 1.13)) in complicated pregnancies. There were no at-risk studies conducted in animals. In human studies only, no differences were observed in normal-growth pregnancies and an increase in at-risk pregnancies (ROM 1.08 (1.02; 1.13) or MD 219 g (65; 374)) and complicated pregnancies (ROM 1.07 (1.03; 1.11) or MD 162 g (69; 255)).

**Figure 2:**
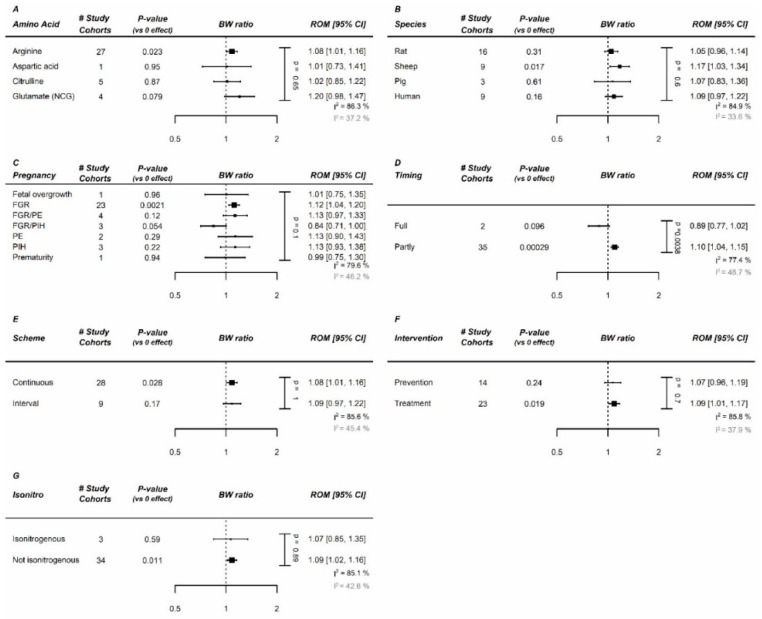
Meta-regression of arginine family on fetal/birth weight (BW) in complicated pregnancies: Meta-regression in complicated pregnancies on (**A**) amino acids (AA), (**B**) species, (**C**) pregnancy complication, (**D**) administration duration, (**E**) administration scheme, (**F**) Intervention type (prevention vs. treatment), and (**G**) isonitrogenous vs. non-isonitrogenous in control arms. NCG and arginine are the most effective AAs, and the largest effect is observed in sheep models of pregnancy complication. Data represent pooled estimates expressed as ratio of means (ROM) with a 95% confidence interval (CI) using a random effect model. Residual I^2^ is shown, and in grey is the residual I^2^ after removal of the outlier Sharky et al. FGR, fetal growth restriction; I^2^, heterogeneity; PE, preeclampsia; PIH, pregnancy-induced hypertension.

**Figure 3:**
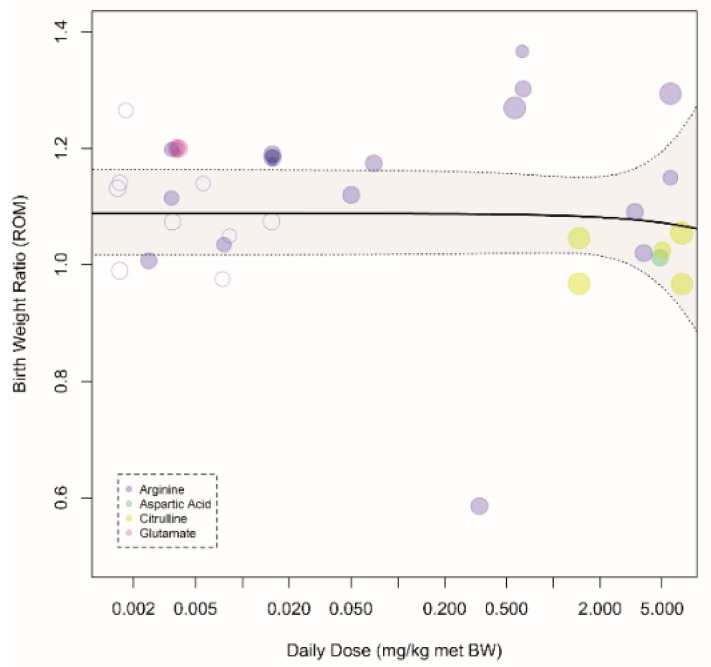
Dose–response curve of prenatal supplementation of the arginine family on fetal/birth weight in complicated pregnancies: Daily dose is expressed as mg per kg metabolic body weight. Open dots indicate human studies, and closed dots indicate animal studies. There is no dose–response relation between prenatal supplementation of amino acids from the arginine family and birth weight ratio (*p*_slope_ = 0.81). An increase of 10% was already reached at the lowest dose.

Within complicated pregnancies, arginine and NCG appeared to be the most effective AAs in the arginine family (Figure 2A). The largest increase was noted in sheep (Figure 2B), in which supplementation consisted of either arginine or NCG. For humans, the effect was also significant (increase of 9%). The effect was comparable between different (induced) pregnancy complications (Figure 2C). AAs from the arginine family appeared to be more effective when supplemented during only one phase of pregnancy, but only two studies supplemented AAs during full pregnancy (Figure 2D). The administration scheme (continuous vs. interval) was not influential (Figure 2E). We observed no effect of a preventive approach versus a therapeutic approach (Figure 2F). Note that, while we did not see clear differences between isonitrogenous vs. non-isonitrogenous control diets, most studies (including all human studies) failed to use isonitrogenous control diets (Figure 2G). Interpretation of the significance of each meta-regression remained unchanged after *p*-value correction for the 7 modifiers (*p* = 0.05/7 = 0.007). A dose–response relation for birth weight was absent with an effective daily dose already reached at the lowest tested dose (Figure 3).

**Figure A2:**
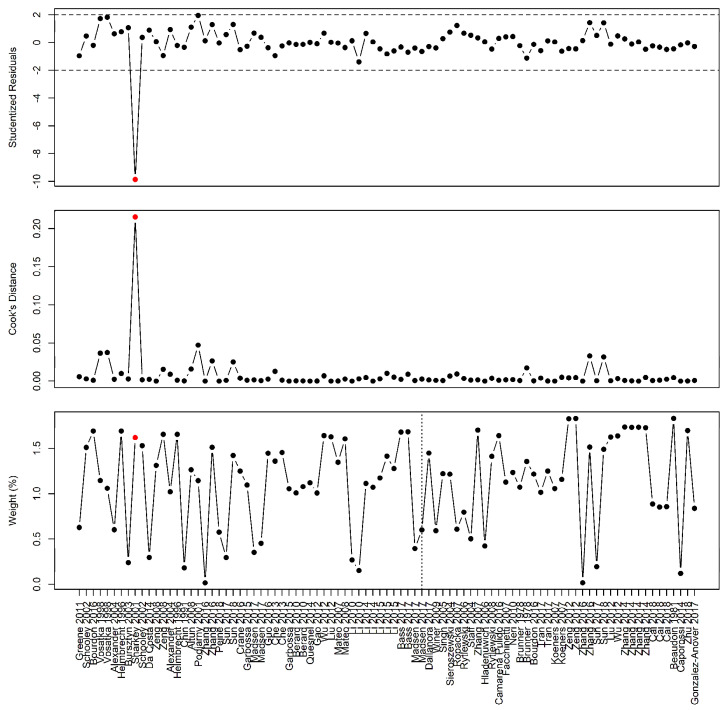
Influential case analysis of studies reporting on arginine family supplementation and fetal/birth weight: A sensitivity analysis revealed Sharkey et al. as an influential case [51].

**Figure A3:**
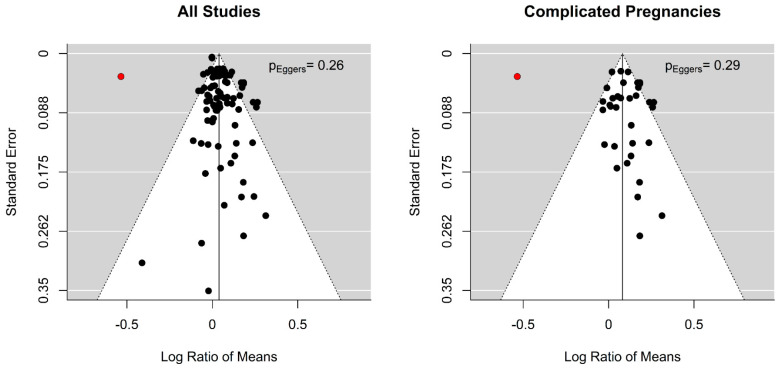
Funnel plot for amino acids of the arginine family and fetal/birth weight in all studies and in studies with only pregnancy complications: These funnel plots and Egger’s regression did not indicate publication bias in all studies or studies in complicated pregnancies. Black dots are the included studies. Sharkey et al., as an influential case, was highlighted by the colour red [51].

The sensitivity analysis identified the rat study by Sharkey et al. [51] as a sensitive case (Figure A2). Removing this study resulted in an increase in body weight by 9% (5; 12) in complicated pregnancy and a reduction of I^2^ from 93% to 77%. Visual inspection of the funnel plot suggested publication bias (Figure A3). However, Eggers regression did not confirm this (*p* = 0.26 for all studies and *p* = 0.29 for studies in complicated pregnancies).

#### 3.3.2. Effect of Prenatal AA in Arginine Family on Maternal Blood Pressure

##### Study Characteristics

The effect on BP was reported in ten rat [34,35,45,48,49,50,51,90,91,92] and six human [78,80,83,89,93,94] studies following supplementation of either arginine (*n* = 15) [34,35,45,48,49,50,51,78,80,83,89,90,91,92,93] or citrulline (*n* = 1 [94] (Table A6). The human studies were performed in France [80], Italy [83], Norway [89], Poland [93], USA [94], and Mexico [78].

##### Meta-Analyses

**Figure A4:**
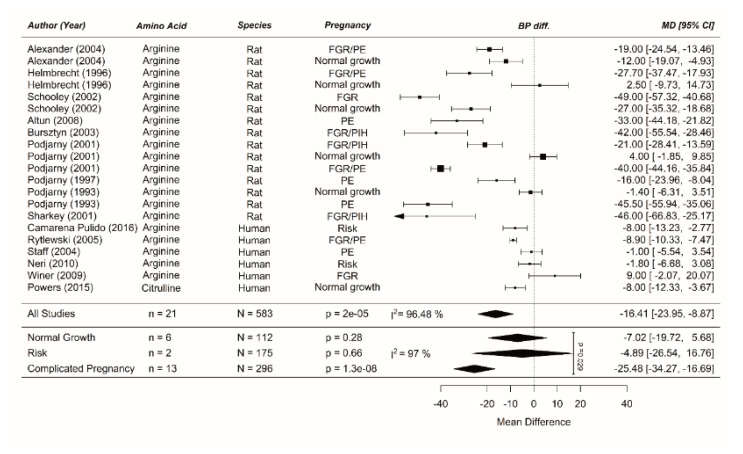
Meta-analysis on prenatal supplementation of arginine on maternal blood pressure: Blood pressure was unaffected in normal-growth pregnancies following arginine supplementation, but was reduced in the risk population and complicated pregnancies. The data is ordered within each amino acid (AA) from smallest to largest animal. Blood pressure difference (BP diff) data represent pooled estimates expressed as mean difference (MD) with 95% confidence interval (CI) using a random effect model. Residual is shown. FGR, fetal growth restriction; I^2^, heterogeneity; PE, preeclampsia; PIH, pregnancy-induced hypertension.

**Figure A5:**
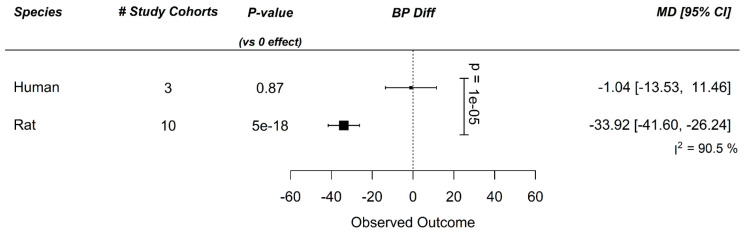
Species meta-regression of amino acids (AA) in arginine family on maternal blood pressure (BP) in pregnancy complications: Meta-regression revealed large interspecies differences, although only two human study cohorts versus seven rat study cohorts reported on the effect of prenatal supplementation of AA of the arginine family in complicated pregnancies. Data represent pooled estimates expressed as a mean difference (MD) with a 95% confidence interval (CI) using a random effect model. I^2^, heterogeneity.

**Figure A6:**
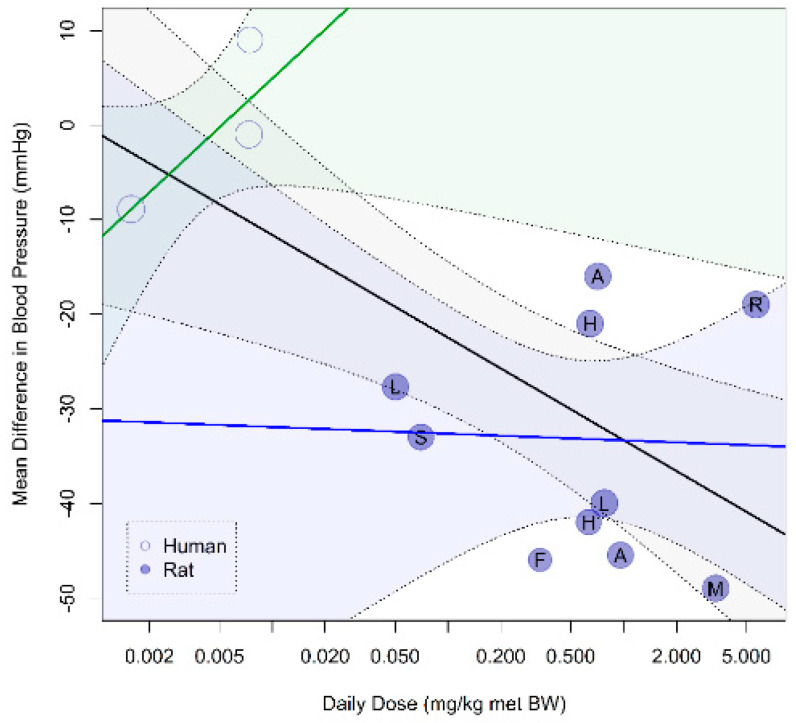
Dose–response curve of prenatal supplementation of arginine family on blood pressure (BP) in complicated pregnancies: Higher doses of arginine result in lower maternal blood pressure in complicated pregnancies (*p*_slope_ = 0.0031). However, this dose–response relation is influenced by an interspecies difference as the higher doses are tested in animal studies and the lowest doses are tested in human studies. Animal models for pregnancy complication included adriamycin nephropathy-induced preeclampsia (A), spontaneous hypertension and heart failure (F), hyperinsulinemic-induced PIH/FGR (H), L-NAME-induced fetal growth restriction/preeclampsia (L), magnesium deficiency-induced fetal growth restriction (M), reduced uterine perfusion pressure-induced fetal growth restriction/preeclampsia (R), and sonic stress-induced preeclampsia (S). Daily dose is expressed as mg per kg metabolic body weight. Open dots indicate human studies, and closed dots indicate animal studies. The black line is drawn for all studies, the yellow line is for animal studies only, and no line is drawn for human studies since only 3 studies were available.

**Figure A7:**
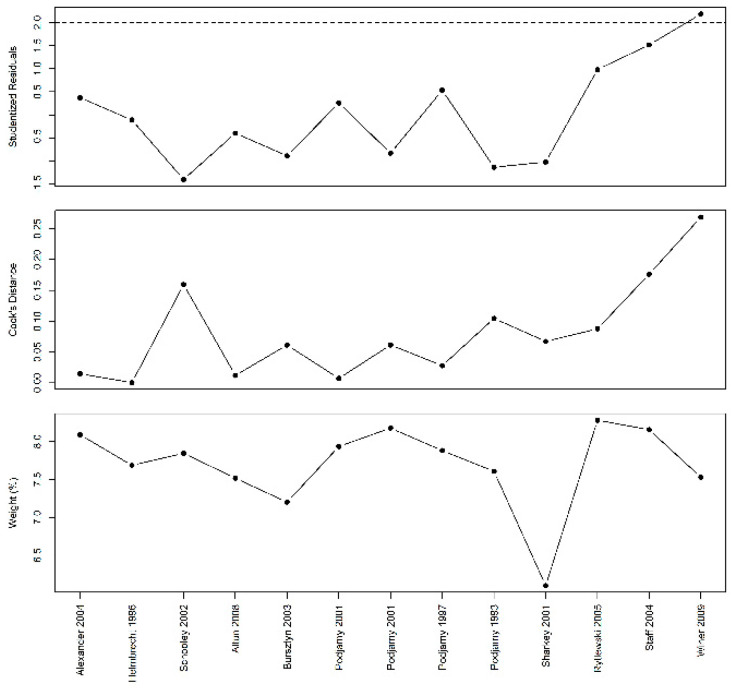
Sensitivity analysis of studies reporting on arginine family supplementation and maternal blood pressure: The sensitivity analysis revealed no clear outlier in studies reporting on arginine family supplementation on maternal blood pressure.

While prenatal supplementation with AAs from the arginine family did not affect BP in normal-growth pregnancies or the risk population, it reduced BPs, with 25 mmHg (−34; −17) in complicated pregnancies (Figure A4). However, this reduction was completely driven by animal studies. In human studies only, no significant BP reduction was observed in either normal-growth (−8 (−21; 5)), at-risk (−5 (−14; 5)), or complicated (−2 (−10; 6)) pregnancies. The BP difference was comparable for the type of BP (mean arterial pressure or systolic BP; data not shown; *p* = NS) [7,31]. Meta-regression showed high interspecies difference in the ten rat and three human study cohorts including pregnancy complications, thus we did not consider further meta-regression analysis rational (Figure A5). In contrast to birth weight outcome, higher doses did result in larger BP differences (Figure A6). Sensitivity analysis did not reveal specific influential cases (Figure A7).

#### 3.3.3. Effect of Prenatal AA in Arginine Family on Prevention of Pregnancy Complications in Risk Populations

##### Study Characteristics

Prevention of pregnancy complications in human risk populations was mostly studied after arginine supplementation (*n* = 8) [78,79,83,85,86,87,95,96] Table A7). All studies reported on the prevalence of SGA. Neri et al. [83] assessed different cut-offs for SGA and showed that, with the same treatment strategy, the risk of developing SGA was lower when a lower cut-off for birth weight was used. This means that especially the more severe FGR was prevented. Only the cut-off of <p10 was included in our meta-analysis. Some of the cohorts also reported lower risk of preterm birth (*n* = 3) [78,79,83] and PE (*n* = 2) [78,79] and no effect on GDM risk (*n* = 1) [78], but there were too few studies to pool data for individual pregnancy complications. The human studies supplementing arginine were performed in Poland (*n* = 4) [85,86,87,95], Mexico (*n* = 2) [83,96], and Italy (*n* = 2) [79,83].

##### Meta-Analyses

**Figure 4:**
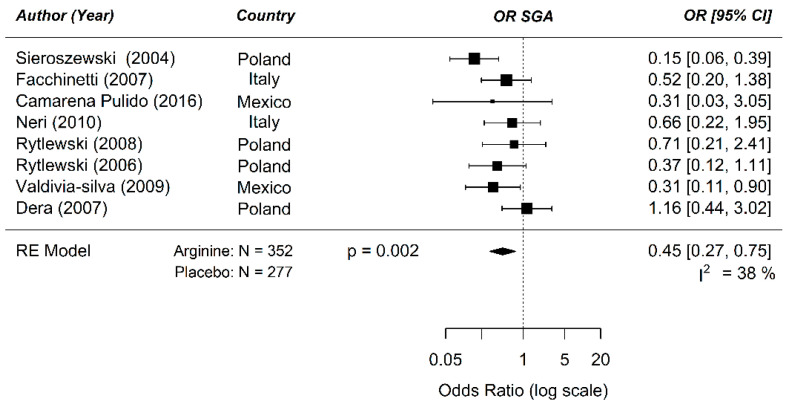
Meta-analysis on the prenatal supplementation of arginine on the development of small for gestational age (SGA) in a human risk population: The odd ratio (OR) for developing SGA in a risk population was 0.45 following arginine supplementation during pregnancy compared to placebo (95% confidence interval (CI) 0.27; 0.75) using a random effect model. Residual I^2^ for heterogeneity is shown.

**Figure A8:**
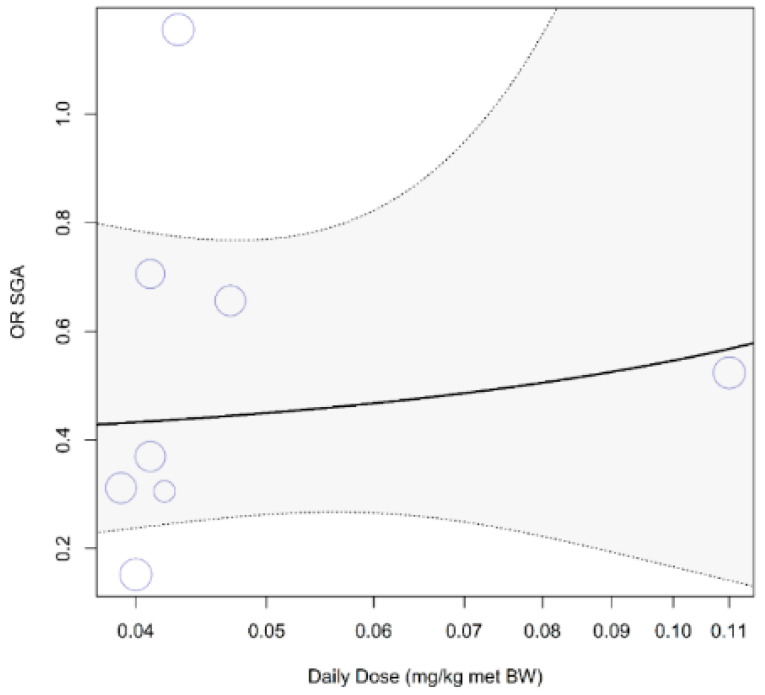
Dose–response curve on the prenatal supplementation of arginine on development of small for gestational age (SGA): The available data points do not show a dose–response relation between odd ratio (OR) of development of SGA and daily arginine dose (*p*_slope_ = 0.73) in a human risk population. Almost all studies clustered at the lower end of the dose spectrum.

The odds ratio for developing SGA in a risk population between prenatal supplementation of arginine and placebo was 0.45 ((0.27; 0.75); *p* = 0.002) (Figure 4). The treatment strategies were similar in these studies (interval, partly, and non-isonitrogenous control diet). Therefore, further meta-regression analysis could not be performed. Based on the sparse data-points, mostly centered around the dose of 0.04 mg/kg, there does not appear to be a clear dose–response relationship (Figure A8).

### 3.4. BCAA

#### 3.4.1. Effect of Prenatal BCAA on Fetal Growth

##### Study Characteristics

Most studies reporting on fetal growth after BCAA supplementation were performed in rats (*n* = 7) [43,97,98,99,100,101,102], with a few in mice (*n* = 1) [103] and pigs (*n* = 2) [104,105]; no human studies were found (Table A5). Leucine was the most investigated BCAA (*n* = 9) [43,97,98,99,100,101,102,103,105], followed by valine (*n* = 4) [43,97,98,104], and isoleucine (*n* = 3) [43,98,99]. The studies performed by Brunner [43], Matsueda [97], and Mori [98] used all three BCAAs.

##### Meta-Analyses

**Figure A9:**
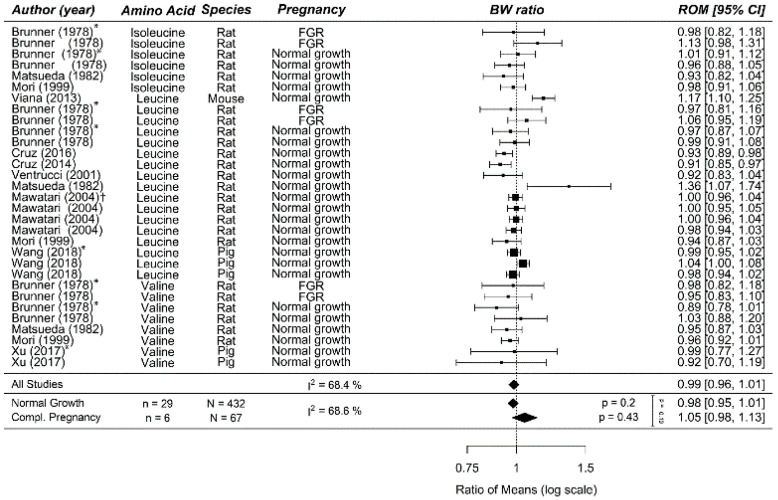
Meta-analysis on prenatal supplementation of branched chain amino acid on fetal/birth weight (BW): Prenatal branched chain amino acid supplementation did not affect birth weight, neither in normal-growth pregnancies nor in complicated pregnancies. Data are ordered within each amino acid (AA) from smallest to largest animal. Data represent pooled estimates expressed as a ratio of means (ROM) with a 95% confidence interval (CI) using a random effect model. Residual I^2^ is shown. Some studies had multiple cohorts and are distinguishable in this figure by the following: * in this upper line, the daily dose is lower compared the next line(s) in increasing order; † the upper two lines are in the lower dose compared to the next two lines, and per dose, the outcomes are separately reported for males first and then females. FGR, fetal growth restriction; I^2^, heterogeneity.

**Figure A10:**
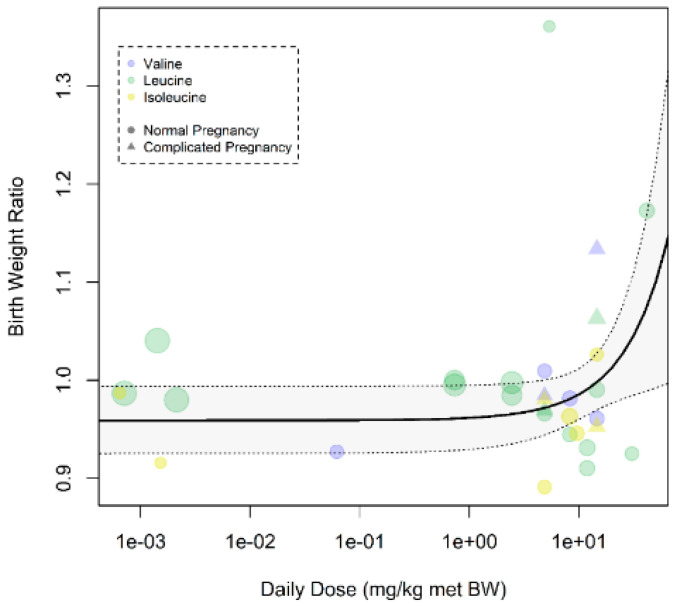
Dose–response curve on prenatal supplementation of branched chain amino acid (BCAA) on fetal/birth weight in all pregnancies: There was a dose–response effect in which the highest doses resulted in a larger improvement of birth weight (*p*_slope_ = 0.006). Only animal studies were included. Daily dose is expressed as mg per kg metabolic body weight.

Prenatal BCAA supplementation did not improve fetal/birth weight in normal-growth (0.98 (0.95; 1.01)) or complicated pregnancy (1.05 (0.98; 1.13), *p* = 0.24, I^2^ = 69%; Figure A9). We were unable to perform meta-regression because Brunner et al. [43] was the only study performed in pregnancy complications (phenylketonuria (PKU)-induced FGR). Brunner et al. [43] tested different dosages and showed that the highest tested dose of leucine and isoleucine were more effective in pregnancy complications. The dose–response curve showed that higher doses of leucine resulted in exponentially higher birth weight in all pregnancies (Figure A10). This effect was less clear for valine or for isoleucine.

**Figure A11:**
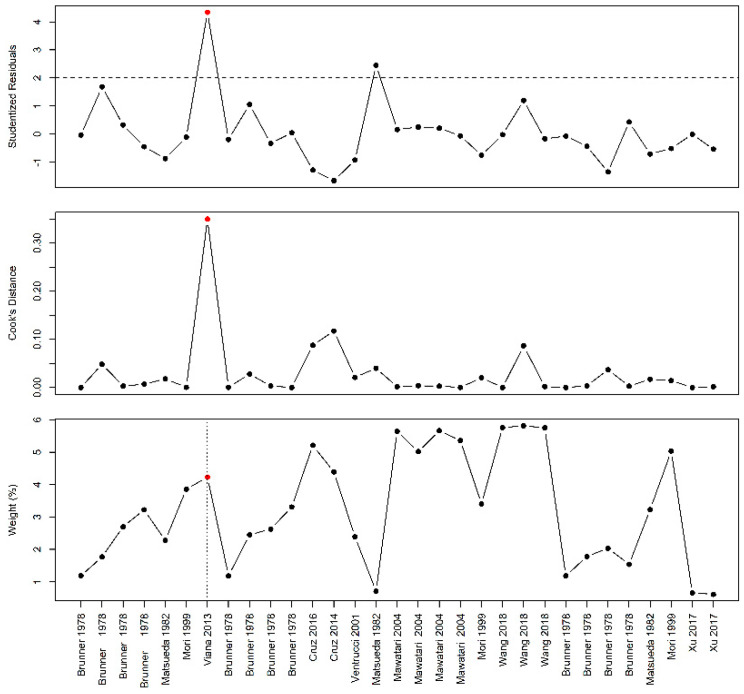
Sensitivity analysis of studies reporting on branched-chain amino acids supplementation and fetal/birth weight: The sensitivity analysis revealed Viana et al. [103] as an influential case.

Sensitivity analysis showed that Viana et al. [103], the only mouse study, was an influential case (Figure A11); removing this study had no significant effect on the pooled effect estimate (0.97 (0.95–0.99); *p* < 0.01), but did reduce I^2^ to 30%.

### 3.5. Methyl Donors

#### 3.5.1. Effect of Prenatal Methyl Donors on Fetal Growth

##### Study Characteristics

We included 30 animal studies (mice *n* = 3 [106,107,108]; rats *n* = 14 [43,97,98,109,110,111,112,113,114,115,116,117,118,119]; guinea pigs *n* = 4 [120,121,122,123]; rabbits = 1 [124]; sheep *n* = 3 [125,126,127]; and cows *n* = 5 [128,129,130,131,132]) and 6 human studies [133,134,135,136,137,138] reporting on fetal growth in response to prenatal methyl donor supplementation. In 16 of these studies, methionine was used [43,97,98,115,116,117,118,119,123,124,125,126,128,129,130,131] while 11 studies supplemented cysteine [106,107,108,113,114,120,121,122,133,134,137] and nine used choline [109,110,111,112,127,132,135,136,138]. Interestingly, considering our hypothesis, only one study used an overgrowth population [113] and only one used an at-risk-of-overgrowth population [132]. The human studies supplementing cysteine were performed in Egypt (*n* = 2) [133,137] and The Netherlands (*n* = 1) [134]. Studies supplementing choline were performed in USA (*n* = 2) [135,136] and South Africa (*n* = 1) [138].

##### Meta-Analyses

**Figure 5:**
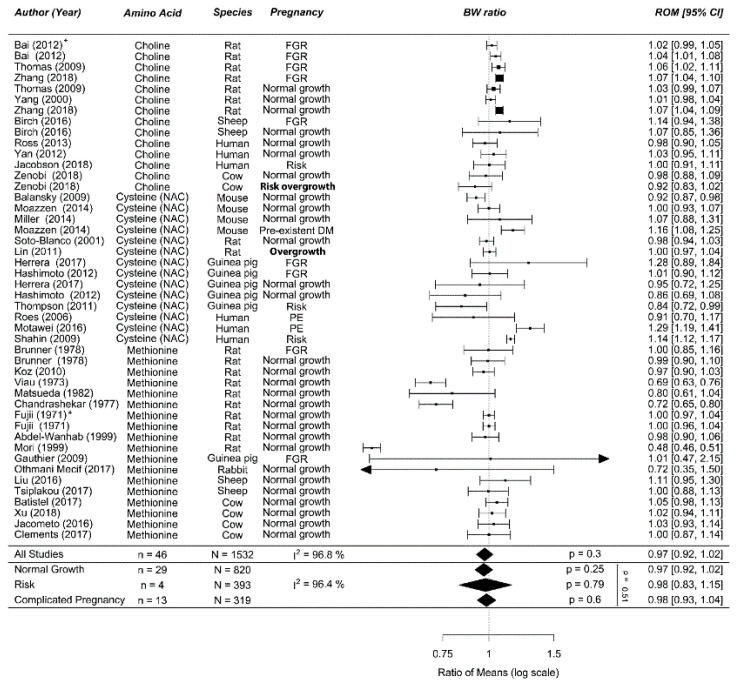
Meta-analysis on prenatal supplementation of methyl donors on fetal/birth weight (BW): Prenatal supplementation of methyl donors did not affect birth weight in normal-growth, risk populations or complicated pregnancies. Data are ordered within each amino acid (AA) from smallest to largest animal. Data represent pooled estimates expressed as a ratio of means (ROM) with a 95% confidence interval (CI) using a random effect model. Residual I^2^ is shown. Only two studies included (risk of) overgrowth as their study population (bold). Some studies had multiple cohorts split up by sex indicated by *, in which the upper line represents male offspring compared to the next line which represents female offspring. FGR, fetal growth restriction; I^2^, heterogeneity; NAC, *N*-acetyl Cysteine; PE, preeclampsia; DM, diabetes mellitus.

**Figure A12:**
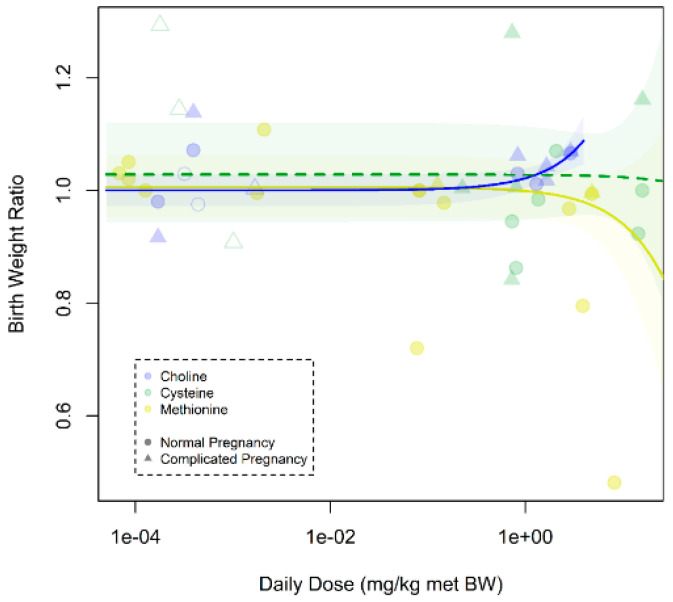
Dose–response curve on prenatal supplementation of methyl donors on fetal/birth weight in all pregnancies: There was a dose–response relationship between birth weight ratio and daily dose of methyl donors (*p*_slope_ = 0.0002). Excessive doses of methionine and cysteine resulted in lower birth weight (methionine *p*_slope_ = 1.09 * 10–5; cysteine *p*_slope_ = 0.16). Daily dose is expressed as mg per kg metabolic body weight. Open dots indicate human studies, and closed dots indicate animal studies.

**Figure A13:**
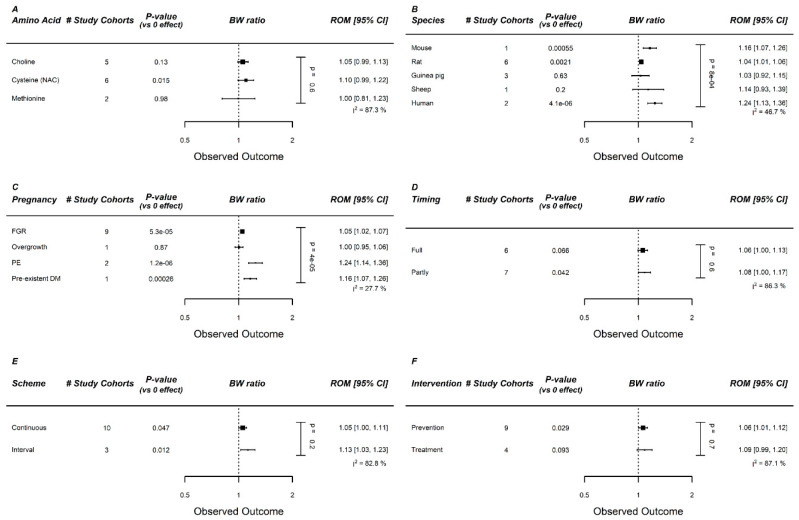
Meta-regression of methyl donors on birth weight (BW): Meta-regression on (**A**) Amino acid (AA), (**B**) species, (**C**) pregnancy complication, (**D**) administration duration, (**E**) administration scheme, and (**F**) intervention type (prevention vs. treatment). No specific methyl donor was identified to be the most optimal. Methyl donor supplementation increased birth weight in human and preeclamptic studies (similar studies). Data represent pooled estimates expressed as a ratio of means (ROM) with a 95% confidence interval (CI) using a random effect model. FGR, fetal growth restriction; I^2^, heterogeneity; PE, preeclampsia; PIH, pregnancy-induced hypertension.

**Figure A14:**
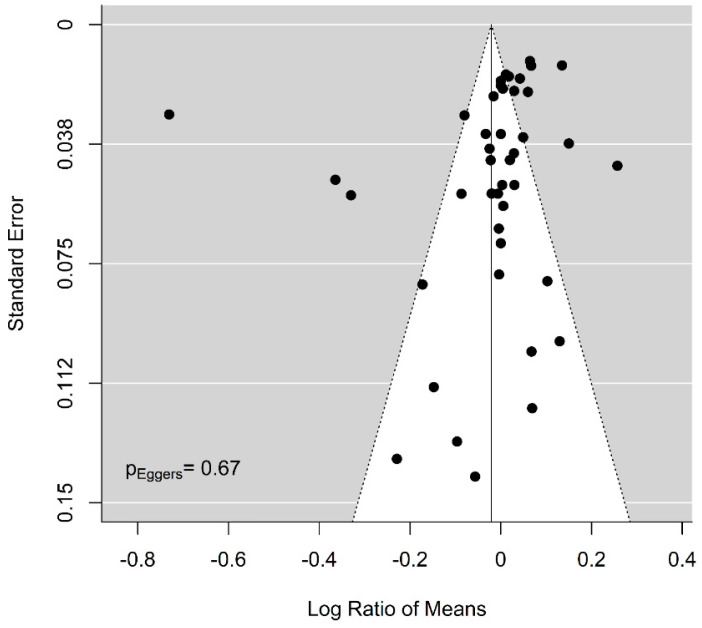
Funnel plot for methyl donors and fetal/birth weight in all studies: The funnel plot and Eggers regression do not indicate publication bias in studies reporting the effect of methyl donor supplementation on fetal or birth weight. Dots are the included studies.

**Figure A15:**
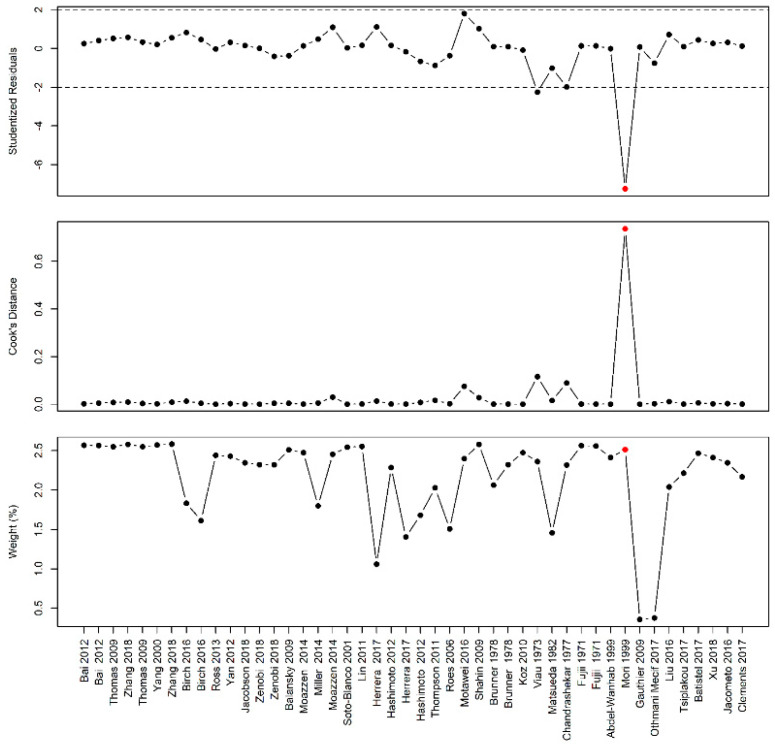
Sensitivity analysis of studies reporting on methyl donor supplementation and fetal/birth weight: The sensitivity analysis revealed Mori et al. [98] as a potential influential case.

Overall, methyl donor supplementation during normal-growth (0.97 (0.92; 1.02)), risk population (0.98 (0.83; 1.15)), or complicated pregnancy (0.98 (0.93; 1.04)) did not alter birth weight (*p* = 0.46; I^2^ = 96%; Figure 5). The two Egyptian studies were the only human studies showing an improvement in birth weight. The dose–response curve showed that higher (excess) doses of methionine and cysteine resulted in a larger reduction of birth weight as was also visible in the forest plot for prenatal methionine in normal-growth pregnancies (Figure A12). Meta-regression showed a lack of effect for all three methyl donors in complicated pregnancies (Figure A13A). Methyl donor supplementation in the two overgrowth (risk) animal studies induced by excess energy and high fat diet failed to influence birth weight [113,132]. However, methyl donors appeared to increase birth weight especially in human pregnancies complicated by PE (Figure A13B,C). Meta-regression did not identify a more effective treatment strategy (Figure A13D–F). Interpretation of the significance of each meta-regression remained unchanged when the *p*-value was corrected for the 6 modifiers (*p* = 0.05/7 = 0.008). There was no clear publication bias visible in the funnel plot (Figure A14), which was supported by Eggers regression (*p* = 0.67). Sensitivity analysis showed that Mori et al. [98] was an influential case (Figure A15). Removing this study had no significant effect on the pooled effect estimate (0.99 (0.95; 1.02), *p* = 0.19, I^2^ = 91%) in normal-growth pregnancies. We speculate that the difference in effect in this study is caused by the high dose of methyl donor.

#### 3.5.2. Effect of Prenatal Methyl Donors on Prevention of Pregnancy Complications in Risk Population

##### Study Characteristics

Prevention of pregnancy complications in a human risk population was studied using choline (*n* = 2, USA and South Africa) [135,138] and cysteine (*n* = 2, Egypt and The Netherlands) [134,137] (Table A7). The included cohorts reported on the prevalence of SGA (*n* = 4) [134,135,137,138], LGA (*n* = 1) [135], preterm birth (*n* = 2) [135,137], PIH (*n* = 2) [135,138], PE (*n* = 1) [138], and GDM (*n* = 2) [135,138]. Three out of four methyl donor studies appeared to reduce the risk of developing SGA [134,137,138], while choline supplementation seemed to worsen GDM and LGA incidence [135,138]. However, there were not enough study cohorts to perform a meta-analysis. The two studies on PIH and the two studies on prematurity showed conflicting results.

### 3.6. Study Quality and Risk of Bias Assessment

**Figure A16:**
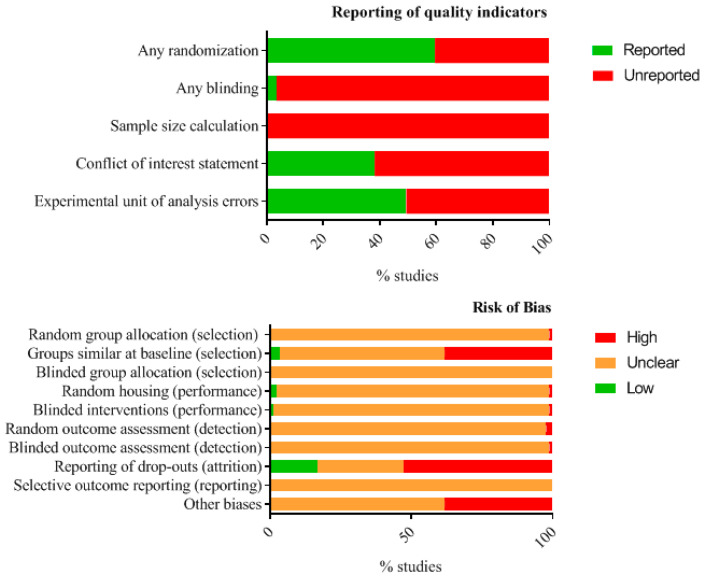
Reporting quality and risk of bias in animal studies: Reporting of key indicators of study quality and risk of bias in animal studies was assessed for all items, but especially blinding, randomization, and sample size calculation scored as unreported, unclear, or high risk.

**Figure A17:**
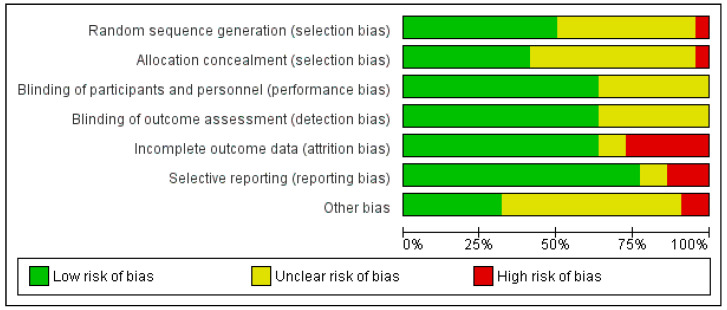
Risk of bias in human studies: Risk of bias assessment in human studies appeared to be very unclear for most items.

**Figure A18:**
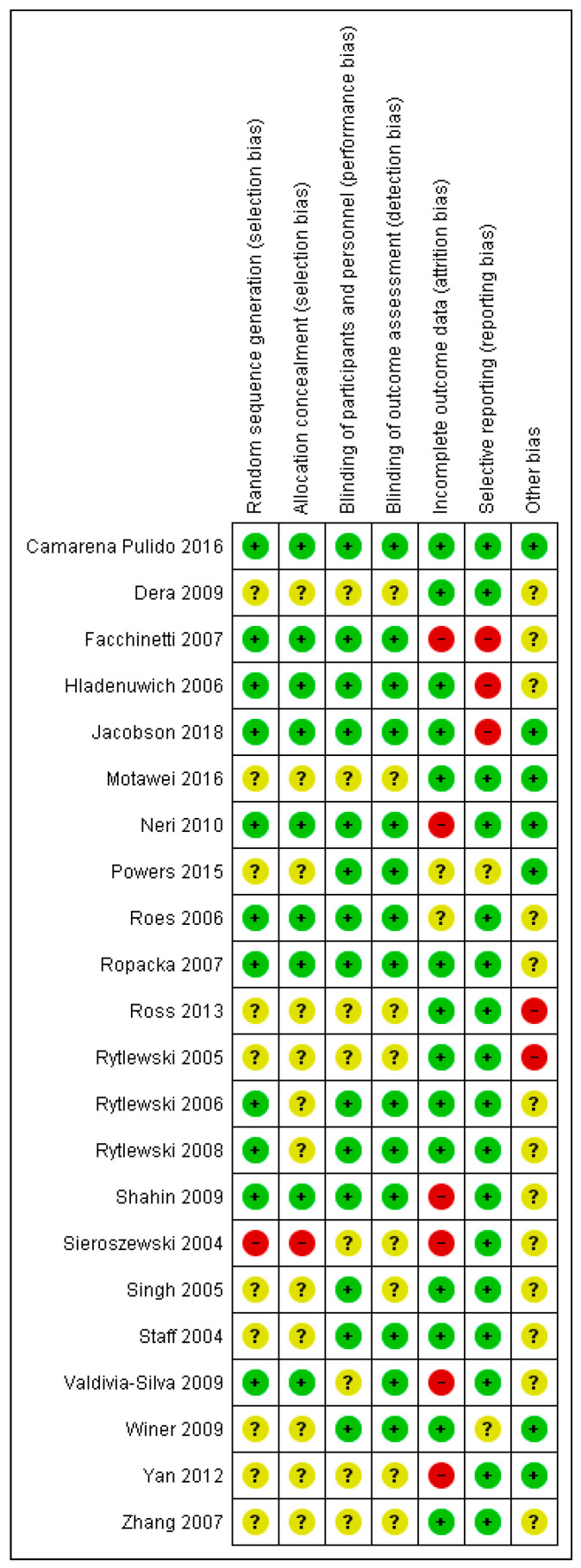
Quality assessment of the included human studies by the Cochrane tool. Quality assessment using the Cochrane risk of bias tool. − = high risk of bias; + = low risk of bias; ? = unclear risk of bias.

The items to determine the risk of bias in animal studies were poorly reported and mostly unclear (Figure A16 and Table A10). The reporting of key indicators of study quality was poor. Especially blinding at any level of the experiment (3%) and power calculations (0%) were hardly reported. The item on experimental unit was important to detect potential statistical errors in the data analysis. In 51% of the studies, it was unclear whether respectively the mothers, or the individual offspring were used as a statistical unit. For risks of bias, a high risk of bias was most often observed for attrition bias (53%), followed by selection bias based on group similarity at baseline (38%). Nearly all studies had an unclear risk of bias for items concerning blinding and randomization (98–100%), because blinding and randomization were either not mentioned at all, or because the methodology used was not described. In human studies, attrition bias constituted the highest risk of bias as well. In addition, the methods used to achieve randomization and blinding were frequently unclear, as was the risk of potential conflict of interest (Figure A17). Only one study had a low risk of bias on all parameters, and the worst score included 3 high risk, 3 unclear risk, and 1 low risk item (Figure A18).

## 4. Discussion

This systematic review and meta-analysis are unique in providing an elaborate overview of prenatal AA supplementation on fetal growth and related pregnancy complications in both humans and animals. Almost all studies focused on the effect of supplementation to target fetal undergrowth. Although 12 of the 14 searched AAs were included, arginine was by far the most studied for all outcome parameters.

### 4.1. Fetal Undergrowth

None of the three AA supplementation groups affected fetal growth in normal-growth pregnancies. Specifically, the arginine family improved fetal growth by 6% in complicated pregnancies. BCAA and methyl donors did not indicate an effect on fetal undergrowth; however, these data were sparse with, for example, only one BCAA study performed in growth-restricted pregnancies and no human studies at all. Within the competent arginine family, arginine and NCG were identified as the most potent, but due to co-linearity in sheep studies, and potential confounding by total nitrogen intake, we cannot conclude this with certainty. The beneficial effect of prenatal arginine supplementation on fetal growth was also reflected by the reduced risk of SGA development in the at-risk human population.

Our observed reduction of BP in hypertensive disorders during pregnancy could prolong pregnancy, thereby improving fetal growth. While there was a strong dose–response curve observed when data across species were combined, no effect or even a potential worsening of BP was observed after supplementation with arginine in women with PE and/or FGR. Arginine might therefore be indicated at low doses to prevent FGR but not as maternal indication to directly treat hypertensive disorders of pregnancy.

The effects evaluated in the present studies might be related to the ability of the placenta to secure adequate essential AA supply towards the fetus, assuming that maternal protein (and nitrogen) intake is of adequate quantity and quality. This would plead for a combined intervention with multiple AAs. Beneficial effects of arginine family supplementation might be mediated through the NO pathway [22]. However, at this stage, we cannot rule out that the effects partially result from arginine stimulating placental nutrient transport or (fetal) protein synthesis through the mTOR pathway [24,139]. This would align with the mTOR-mediated alleviation of FGR observed after leucine supplementation [140].

### 4.2. Fetal Overgrowth

We hypothesized that methyl donors could potentially normalize overgrowth. Unfortunately, only two studies used methyl donor supplementation (and one used arginine) in overgrowth (risk) pregnancies, leaving the answer to the research question inconclusive. Methionine at (very) high doses reduced fetal growth in normal-growth pregnancies. This is potentially due to reduced maternal food intake and a reduction in ovarian steroidogenic pathway activity that could be rescued by administration of exogenous estrone and progesterone [98,116,119]. However, even in rats administered estrone and progesterone, fetal weight was still reduced compared to pair-fed controls, so additional mechanisms may be involved [116,119]. Several human studies have also reported side effects of methionine at extremely high levels [141].

Very little research was performed in diabetic pregnancies regarding the possible effect of AA supplementation on glucose and insulin levels. However, oral administration of choline prior to and during pregnancy in mouse models of maternal obesity has been reported to reduce fetal overgrowth [26,142], which is a common complication in diabetic pregnancy.

### 4.3. Strengths and Limitations

The major strength of this meta-analysis involves integration of data across species. This relatively novel but increasingly used methodology has been shown to be of great value to improve translation from animal studies to humans in several fields since (1) they provide insight on the safety of interventions because of the larger range of dosages, (2) they aid in determining factors influencing the effect size, (3) they reveal biases thus leading to less misinterpretation, and (4) they clarify differences in design between animal and human studies [143,144]. For instance, we previously showed that a large RCT might not have not observed benefits of a treatment due to underdosage [31]. In this integrated meta-analysis, we additionally combined different groups of AAs that act through different pathways, but included only oral supplementation, and different dosages, all to get one step closer to the bedside. This was a valuable approach for the arginine family, but the relative scarcity of studies performed in complicated pregnancy settings compared to normal-growth ones for the BCAAs and methyl donors limited our ability to draw conclusions about which AAs would be most efficient.

Higher heterogeneity in this integrated type of meta-analysis compared to clinical meta-analyses is inevitable due to the inclusion of different experimental designs. Of note, the aim of a meta-analysis of animal studies alone or combined with human studies is not to pinpoint the effect estimated to directly drive clinical practice. Rather, their goal is to investigate factors influencing treatment efficacy, by determining sources of heterogeneity. As such, high heterogeneity provides the chance to explore its source, and the results generate new hypotheses on how to improve efficacy of the intervention or design of future (human) studies. However, the relatively high heterogeneity in our meta-analysis could not always be fully explained by the performed meta-regression. Socio-economic status taken as a surrogate for baseline nutritional status could influence in particular the results of human supplementation, but included studies were performed either in countries with a similar socio-economic status/ethnics division or that did not have a different impact on effect size. Furthermore, animal models represent a part of a complex syndrome and could influence the results, with our main concern regarding studies supplementing with arginine in compromised animal models by a manipulated NO-pathway However, we could not identify an effect on birth weight or blood pressure when excluding studies using L-NAME-induced animal models.

Our risk of bias tool revealed that most human and animal studies failed to report on quality items or risk of bias items. The unclear risk of bias must be taken into account when interpreting the results (of the individual studies and of our meta-analysis). As we did not exclude any studies based on their risk of bias score, this may have contributed to the high heterogeneity (although it was unclear to what extent, as they were not reported). One study [51] could be considered an influential case in our meta-analysis, since removal of this study would result in a significant drop in I^2^ value in both the overall meta-analysis and meta-regressions on the effect of arginine family supplementation and birth weight. However, we could not find any reason for the apparent atypical result found in this study (and have therefore not excluded this study from analysis).

Furthermore, fetal/birth weight is an interesting direct pregnancy outcome, but it does not necessarily correlate with other important obstetric, neonatal, and developmental programming outcomes related to improved long-term health. Hence, BCAA or methyl donors could have no effect on birth weight while still having beneficial or adverse developmental programming effects [145,146]. This was beyond the scope of our meta-analysis.

### 4.4. Perspectives

Overall, this systematic review gives a broad overview of the reported effects of oral prenatal AA supplementation on fetal growth and related pregnancy outcomes. We conclude that none of the AA groups had any adverse effects on fetal growth at low doses. Supplementation with AAs from the arginine family improved birth weight in complicated pregnancies, and reduced risk of SGA development in a human risk population. However, the potency on maternal BP was less clear and the arginine family might not be indicated as maternal treatment for hypertensive disorders of pregnancies. Based on this systematic review and meta-analysis, we formed recommendations for future research, which are summarized in Table 1. We plead for better and well-controlled study designs by using the most suitable study population and animal models, isonitrogenous control diets, and similar baseline nutritional state. In addition, the risk of bias could be reduced by a preplanned protocol describing the intended outcomes, and blinding and randomization methods. Supplementation of BCAA and methyl donors requires more research in animal studies to subsequently determine their potential on fetal growth, blood glucose, and HOMA-IR in models of pregnancies complicated by GDM or fetal overgrowth. The optimal combination of several AAs complemented with potential co-factors should be determined in future research. However, the beneficial effects that this review presents encourages a human RCT on supplementation of arginine family members, with an isonitrogenous control diet, to treat and prevent fetal growth restriction.

## Figures and Tables

**Table 1:** Recommendations for future research.

Type of AA	Recommendation
Arginine family	Large well-controlled RCTs with arginine or NCG as the most potential AA within the arginine family in pregnancies with fetal growth restriction and risk populations
BCAA	Fetal growth effect in animal models of pregnancy complications, especially linked to fetal undergrowth
Methyl donors	Effect on fetal growth, blood glucose, and HOMA-IR in animal models of pregnancies complicated by GDM or fetal overgrowth
General	Well-defined phenotypes of the target population and animal models for specific pregnancy outcomes Studies to the optimal combination of (low doses of) several AAs, potentially with other co-factors depending on severity of growth deviation Studies should always include isonitrogenous control diets

AA, amino acid; BCAA, branched chain amino acids; GDM, gestational diabetes mellitus; NCG, *N*-Carbamylglutamate; RCT, randomized controlled trial.

## Appendix A

### Appendix A.1. Expanded Methods

#### Appendix A.1.1. Study Selection: Inclusion and Exclusion Criteria

The screening of hits was conducted by two independent investigators, first based on their title and abstract and, subsequently, eligible articles were screened for final inclusion based on their full-text. A third investigator was consulted when consensus was not reached.

#### Appendix A.1.2. Exclusion Criteria

Studies were excluded in cases of combined intervention (e.g., supplementation with two or more AAs in the treatment arm), other administration routes than oral, intervention not during pregnancy, pre-conceptional administration, supplementation other than the 14 specified AAs, no control treatment group present, non-mammals, no outcome of interest as previously mentioned, irretrievable full-text or meeting abstract irretrievable, or if the research articles on the studies did not contain unique primary data.

#### Appendix A.1.3. Data-Extraction

Data was extracted on study characteristics, including species, strain, animal model, pregnancy complication, and maternal weight. Pregnancies complicated by placental insufficiency were labelled as one or a combination of the following: fetal growth restriction (FGR), preeclampsia (PE), or pregnancy-induced hypertension (PIH). Regarding supplementation strategy, we extracted data on the dose in grams per kg body weight per day, the duration of supplementation, the timing during pregnancy (partly or full), the administration scheme (continuous versus interval), the intervention type (prevention or treatment), and whether an isonitrogenous control diet was provided. Maternal weight was used to calculate the dose in grams per kg body weight per day, and maternal weight was estimated when not provided. For birth/fetal weight, the number of offspring and sex was also extracted. For maternal BP, the method and type of measurement were extracted. We also extracted whether BP measurements were performed under stressful condition; in humans, whether it concerned a 24 h or office BP measurement; and in animals, whether BP was measured under restrained or unrestrained conditions.

When data was only presented graphically, we used a graph digitizer to extract the data (http://arohatgi.info/WebPlotDigitizer/). We contacted corresponding authors once per email in case of missing data. SEM and pooled SEM were converted to SDs. The Hozo formula was used to estimate the mean and SD when the median was reported [147].

#### Appendix A.1.4. Amendments to Protocol

The following amendments to the review protocol were made post hoc: dose–response curves, meta-regression in type of pregnancy complication, and isonitrogenous versus non-isonitrogenous control diet in arginine family. We also changed the categories early, mid, late, and full gestational to partly vs. full gestational, because most studies reported overlapping parts during pregnancy (e.g., early-mid) which resulted in multiple categories with only one study per category. Also, pregnancy “trimesters” and the stage of development were difficult to compare between species. We extracted data on basal protein intake, but we could not perform our initial planned meta-regression. Since the cut-off of when basal protein intake is too low differs per species, and the individual intake and maternal weight (gain) were not reported, we could not convert the extracted data into a unit of measurement that we could pool.

#### Appendix A.1.5. Adjustments Made to the Risk of Bias Tools

To the SYRCLE tool, we added the reporting item whether the correct experimental unit was used. For the item of comparable baseline characteristics, we assessed (1) whether induction of the animal model occurred at the same gestational age, (2) whether the age or weight of pregnant animal was similar (<10% difference), and (3) whether parity (virgin or multiple pregnancies) was similar between groups. Other risks of bias within the Cochrane tool entailed a statement of no conflict of interest.

### Appendix A.2. Expanded Results

We also searched each paper for data on protein intake and body composition. Body composition was never mentioned; therefore, meta-regression was not possible. Below, we describe, in the few studies where these data were available, the effect of prenatal supplementation of AA from the arginine family, BCAA, and methyl donors on maternal weight gain and blood glucose levels.

#### Appendix A.2.1. Effect of Prenatal AA in Arginine Family on Maternal Weight Gain and Blood Glucose Levels

Gestational weight gain was reported in 23 animal studies (1 mouse [33], 10 rat [36,37,43,44,45,46,49,90,91,148], 3 sheep [53,54,55], and 9 pig/swine [58,59,66,67,69,74,75,76,149]) and only 1 human study (Poland) [95] (Table A8). Again, the most studied amino acid was arginine (*n* = 19) [33,36,37,44,45,46,49,53,54,55,58,59,69,74,75,76,90,91,95], but also glutamate (*n* = 5) [54,55,66,148,149], citrulline (*n* = 1) [44], and glutamine (*n* = 1) [67] were studied. Thirteen studies were conducted in normal-growth pregnancies only [33,37,46,58,59,66,67,69,74,75,76,148,149], seven were conducted in complicated pregnancies only [36,44,45,49,53,54,55], three studies had both normal-growth and complicated pregnancy arms [43,90,91], and one study was conducted in a risk population [95]. Of the 10 studies including complicated pregnancies, only 4 showed an increase in gestational weight gain [36,54,55]. As none of these studies included any normal-growth pregnancies, it is not possible to see whether the increase meant a normalization of gestational weight gain. The other 6 studies reported no significant effects on gestational weight gain. One of the two studies in at-risk cohorts showed a positive effect as well [49].

None of the studies reported HOMA-IR levels, and only seven animal studies reported blood glucose levels: two rat [45,49], one sheep [57], and three pig studies [67,74,77] (Table A9). All studies supplemented with arginine and two studies had an extra cohort with NCG supplementation [57,77]. None of the studies in normal-growth pregnancies (*n* = 3), complicated (*n* = 2), or at-risk pregnancies (*n* = 1) reported significant effects of arginine supplementation on maternal blood glucose levels.

#### Appendix A.2.2. Effect of Prenatal BCAA on Maternal Weight Gain and Glucose Levels

Maternal weight gain was reported in four rat studies; of these, Brunner [43], Matsueda [97], and Mori [98] tested in different treatment arms the effects of valine, leucine, and isoleucine and Ventrucci [101] studied only leucine supplementation (Table A8). Almost all studies included normal-growth pregnancies in which no effect was observed. The one study including pregnancies complicated by FGR found some effects in high-dose groups: a reduction in gestational weight gain following valine, an increase following leucine, and no significant effects of isoleucine [43]. Glucose levels could only be extracted from one study, using leucine supplementation in normal-growth pregnant rats [150]. In this study, leucine supplementation increased maternal blood glucose levels. As for the arginine family and maternal weight gain, we were unable to pool the data.

#### Appendix A.2.3. Effect of Prenatal Methyl Donors on Maternal Weight Gain and Glucose Levels

We included 9 animal studies on maternal weight gain, which were performed in mice (*n* = 1) [108], rats (*n* = 6) [43,97,98,109,112,114], sheep (*n* = 1) [125], and cows (*n* = 1) [151], and 3 human studies [135,136,138] (Table A8). Most studies used choline (*n* = 6) [109,112,135,136,138,151], but also methionine (*n* = 4) [43,97,98,125] and cysteine (*n* = 2) [108,114] were investigated. Nine studies used normal-growth pregnancies [97,98,108,114,125,135,136,151], one of which also included a complicated pregnancy arm [43]. Two studies used complicated pregnancies [109,112], and one study was conducted in an at-risk population [138]. None of the studies in complicated or at-risk pregnancies reported an effect.

We included four animal studies reporting on maternal blood glucose level in response to methyl donor supplementation [107,113,132,152] (Table A9). Maternal blood glucose levels remained in the same range following choline supplementation in two cow studies including normal-growth, FGR, or overgrowth risk [132,152]. However, the two cysteine studies reported significant increases in maternal blood glucose levels in a streptozotocin-induced pre-existent diabetes mellitus (DM) type 1 mice model [107] and an overgrowth risk rat model using high fat diet [113].

## Appendix B

**Table A1:** Search terms in Pubmed.

14 amino acids AND administration	(“Arginine/administration and dosage” [Mesh] OR “Arginine/adverse effects” [Mesh] OR “Arginine/deficiency” [Mesh] OR “Arginine/drug effects” [Mesh] OR “Arginine/physiology” [Mesh] OR “Arginine/pharmacology” [Mesh] OR “Arginine/therapeutic use” [Mesh] OR “Arginine/therapy” [Mesh] OR “Leucine/administration and dosage” [Mesh] OR “Leucine/adverse effects” [Mesh] OR “Leucine/deficiency” [Mesh] OR “Leucine/drug effects” [Mesh] OR “Leucine/physiology” [Mesh] OR “Leucine/pharmacology” [Mesh] OR “Leucine/therapeutic use” [Mesh] OR “Isoleucine/administration and dosage” [Mesh] OR “Isoleucine/adverse effects” [Mesh] OR “Isoleucine/deficiency” [Mesh] OR “Isoleucine/drug effects” [Mesh] OR “Isoleucine/physiology” [Mesh] OR “Isoleucine/pharmacology” [Mesh] OR “Isoleucine/therapeutic use” [Mesh] OR “Valine/administration and dosage” [Mesh] OR “Valine/adverse effects” [Mesh] OR “Valine/deficiency” [Mesh] OR “Valine/drug effects” [Mesh] OR “Valine/physiology” [Mesh] OR “Valine/pharmacology” [Mesh] OR “Valine/therapeutic use” [Mesh] OR “Valine/therapy” [Mesh] OR “Cysteine/administration and dosage” [Mesh] OR “Cysteine/adverse effects” [Mesh] OR “Cysteine/deficiency” [Mesh] OR “Cysteine/drug effects” [Mesh] OR “Cysteine/physiology” [Mesh] OR “Cysteine/pharmacology” [Mesh] OR “Cysteine/therapeutic use” [Mesh] OR “Cysteine/therapy” [Mesh] OR “Methionine/administration and dosage” [Mesh] OR “Methionine/adverse effects” [Mesh] OR “Methionine/deficiency” [Mesh] OR “Methionine/drug effects” [Mesh] OR “Methionine/physiology” [Mesh] OR “Methionine/pharmacology” [Mesh] OR “Methionine/therapeutic use” [Mesh] OR “Methionine/therapy” [Mesh] OR “Glutamic Acid/administration and dosage” [Mesh] OR “Glutamic Acid/adverse effects” [Mesh] OR “Glutamic Acid/deficiency” [Mesh] OR “Glutamic Acid/drug effects” [Mesh] OR “Glutamic Acid/pharmacology” [Mesh] OR “Glutamic Acid/physiology” [Mesh] OR “Glutamic Acid/therapeutic use” [Mesh] OR “Glutamine/administration and dosage” [Mesh] OR “Glutamine/adverse effects” [Mesh] OR “Glutamine/deficiency” [Mesh] OR “Glutamine/drug effects” [Mesh] OR “Glutamine/pharmacology” [Mesh] OR “Glutamine/physiology” [Mesh] OR “Glutamine/therapeutic use” [Mesh] OR “Glutamine/therapy” [Mesh] OR “Citrulline/administration and dosage” [Mesh] OR “Citrulline/adverse effects” [Mesh] OR “Citrulline/deficiency” [Mesh] OR “Citrulline/drug effects” [Mesh] OR “Citrulline/pharmacology” [Mesh] OR “Citrulline/physiology” [Mesh] OR “Citrulline/therapeutic use” [Mesh] OR “Asparagine/administration and dosage” [Mesh] OR “Asparagine/adverse effects” [Mesh] OR “Asparagine/deficiency” [Mesh] OR “Asparagine/drug effects” [Mesh] OR “Asparagine/pharmacology” [Mesh] OR “Asparagine/physiology” [Mesh] OR “Asparagine/therapeutic use” [Mesh] OR “Asparagine/therapy” [Mesh] OR “Asparagine/administration and dosage” [Mesh] OR “Asparagine/adverse effects” [Mesh] OR “Asparagine/deficiency” [Mesh] OR “Asparagine/drug effects” [Mesh] OR “Asparagine/pharmacology” [Mesh] OR “Asparagine/physiology” [Mesh] OR “Asparagine/therapeutic use” [Mesh] OR “Asparagine/therapy” [Mesh] OR “Aspartic Acid/administration and dosage” [Mesh] OR “Aspartic Acid/adverse effects” [Mesh] OR “Aspartic Acid/deficiency” [Mesh] OR “Aspartic Acid/drug effects” [Mesh] OR “Aspartic Acid/pharmacology” [Mesh] OR “Aspartic Acid/physiology” [Mesh] OR “Aspartic Acid/therapeutic use” [Mesh] OR “Aspartic Acid/therapy” [Mesh] OR “Proline/administration and dosage” [Mesh] OR “Proline/adverse effects” [Mesh] OR “Proline/deficiency” [Mesh] OR “Proline/drug effects” [Mesh] OR “Proline/pharmacology” [Mesh] OR “Proline/physiology” [Mesh] OR “Proline/therapeutic use” [Mesh] OR “Ornithine/administration and dosage” [Mesh] OR “Ornithine/adverse effects” [Mesh] OR “Ornithine/deficiency” [Mesh] OR “Ornithine/drug effects” [Mesh] OR “Ornithine/pharmacology” [Mesh] OR “Ornithine/physiology” [Mesh] OR “Ornithine/therapeutic use” [Mesh] OR “Choline/administration and dosage” [Mesh] OR “Choline/adverse effects” [Mesh] OR “Choline/deficiency” [Mesh] OR “Choline/drug effects” [Mesh] OR “Choline/pharmacology” [Mesh] OR “Choline/physiology” [Mesh] OR “Choline/therapeutic use” [Mesh] OR “Choline/therapy” [Mesh]) OR ((“Arginine” [Mesh] OR Arginine [tiab] OR L-Arginine [tiab] OR “Leucine” [Mesh] OR “Isoleucine” [Mesh] OR Leucine [tiab] OR L-Leucine [tiab] OR Leucin [tiab] OR Isoleucine [tiab] OR Isoleucin [tiab] OR Alloisoleucine [tiab] OR Alloisoleucin [tiab] OR “Valine” [Mesh] OR Valine [tiab] OR L-Valine [tiab] OR Valsartan [tiab] OR Valerate [tiab] OR “Cysteine” [Mesh] OR Cysteine [tiab] OR L-Cysteine [tiab] OR Cysteinate [tiab] OR Acetylcysteine [tiab] OR Carbocysteine [tiab] OR Cysteinyldopa [tiab] OR Cystine [tiab] OR cystein [tiab] OR cysthion [tiab] OR Selenocysteine [tiab] OR “Methionine” [Mesh] OR Methionine [tiab] OR L-Methionine [tiab] OR Liquimeth [tiab] OR Pedameth [tiab] OR Formylmethionine [tiab] OR Racemethionine [tiab] OR Adenosylmethionine [tiab] OR Selenomethionine [tiab] OR Vitamin U [tiab] OR acimetion [tiab] OR cotameth [tiab] OR lobamine [tiab] OR menin [tiab] OR menine [tiab] OR meonine [tiab] OR methiolate [tiab] OR methionin [tiab] OR methiotrans [tiab] OR methnine [tiab] OR methurine [tiab] OR metione [tiab] OR methidin [tiab] OR neutrodor [tiab] OR oradash [tiab] OR urosamine [tiab] OR Methyl Donor [tiab] OR “Glutamic Acid” [Mesh] OR glutamic acid [tiab] OR glutamate [tiab] OR MSG [tiab] OR vestin [tiab] OR “aminoglutaric acid” [tiab] OR “aminopentanedioic acid” [tiab] OR acidogen [tiab] OR acidoride [tiab] OR acidothym [tiab] OR acidulin [tiab] OR aciglumin [tiab] OR aciglut [tiab] OR aclor [tiab] OR antalka [tiab] OR flanithin [tiab] OR gastuloric [tiab] OR glusate [tiab] OR glutadox [tiab] OR glutamidin [tiab] OR “glutamin acid” [tiab] OR “glutaminic acid” [tiab] OR glutaminol [tiab] OR glutan [tiab] OR glutansin [tiab] OR glutasin [tiab] OR glutaton [tiab] OR hydrionic [tiab] OR hypochylin [tiab] OR levoglutamate [tiab] OR “levoglutamic acid” [tiab] OR muriamic [tiab] OR pepsdol [tiab] OR “Glutamine” [Mesh:NoExp] OR glutamine [tiab] OR “aminoglutaramic acid” [tiab] OR acutil [tiab] OR “adamin G” [tiab] OR glumin [tiab] OR glutamin [tiab] OR levoglutamide [tiab] OR levoglutamine [tiab] OR nutrestore [tiab] OR “Citrulline” [Mesh] OR citrulline [tiab] OR “ureidopentanoic acid” [tiab] OR ureidonorvaline [tiab] OR “ureidovaleric acid” [tiab] OR citrullin [tiab] OR carbamylornithine [tiab] OR “Asparagine” [Mesh] OR asparagine [tiab] OR asparagin [tiab] OR “aminosuccinamic acid” [tiab] OR “Aspartic Acid” [Mesh:NoExp] OR “D-Aspartic Acid” [Mesh] OR “Potassium Magnesium Aspartate” [Mesh] OR “aspartic acid” [tiab] OR aspartate [tiab] OR Magnesiocard [tiab] OR Mg-5-Longoral [tiab] OR Mg 5 Longoral [tiab] OR Mg5 Longoral [tiab] OR panangin [tiab] OR astra 2045 [tiab] OR “aminosuccinic acid” [tiab] OR “asparagic acid” [tiab] OR asparaginate [tiab] OR “asparaginic acid” [tiab] OR aspartyl [tiab] OR aspatofort [tiab] OR “levoaspartic acid” [tiab] OR “Proline” [Mesh:NoExp] OR proline [tiab] OR prolin [tiab] OR levoproline [tiab] OR “pyrrolidinecarboxylic acid” [tiab] OR pyrrolidine carboxylate [tiab] OR “Ornithine” [Mesh:NoExp] OR ornithine [tiab] OR ornithin [tiab] OR “Diaminopentanoic Acid” [tiab] OR “diaminovaleric acid” [tiab] OR “Choline” [Mesh:NoExp] OR choline [tiab] OR bursine [tiab] OR vidine [tiab] OR fagine [tiab] OR trimethylammonium hydroxide [tiab] OR amonita [tiab] OR bilineurine [tiab] OR biocholine [tiab] OR biocolina [tiab] OR cholin [tiab] OR hepacholine [tiab] OR laevocholine [tiab] OR levocholine [tiab] OR lipotril [tiab] OR luridine [tiab] OR sincaline [tiab] OR urocholine [tiab]) AND (“Dietary Supplements” [Mesh] OR “Administration, Oral” [Mesh] OR administration * [tiab] OR administer * [tiab] OR dose [tiab] OR doses [tiab] OR dosage [tiab] OR treatment [tiab] OR treated [tiab] OR supplement * [tiab] OR diet [tiab] OR diets [tiab] OR dietary [tiab] OR intake [tiab] OR intakes [tiab] OR consumption [tiab] OR consumptions [tiab] OR consume [tiab] OR nutraceutical * [tiab] OR nutriceutical * [tiab] OR therapy [tiab] OR therapies [tiab]))
AND	AND
Healthy pregnancy OR Complicated pregnancy	“Pregnancy” [Mesh] OR “gravidity” [Mesh] OR “Fetus” [Mesh] OR Pregnancy [tiab] OR Pregnancies [tiab] OR Pregnant [tiab] OR Gestation [tiab] OR Gestations [tiab] OR Gestational [tiab] OR gravidity [tiab] OR gravidities [tiab] OR gravid [tiab] OR fetus [tiab] OR foetus [tiab] OR fetal [tiab] OR foetal [tiab] OR childbearing [tiab] OR “child bearing” [tiab] OR “Fetal Growth Retardation” [Mesh] OR “Infant, Low Birth Weight “ [Mesh] OR “Infant, Premature” [Mesh] OR “Premature Birth” [Mesh] OR FGR [tiab] OR Intrauterine growth retardation [tiab] OR Intra-uterine growth retardation [tiab] OR Intrauterine growth restriction [tiab] OR Intra-uterine growth restriction [tiab] OR IUGR [tiab] OR Small for Gestational Age [tiab] OR SGA [tiab] OR Low birth weight [tiab] OR Premature baby [tiab] OR Pre-mature baby [tiab] OR Preterm baby [tiab] OR Pre-term baby [tiab] OR Premature babies [tiab] OR Pre-mature babies [tiab] OR Preterm babies [tiab] OR Pre-term babies [tiab] OR Premature child [tiab] OR Pre-mature child [tiab] OR Preterm child [tiab] OR Pre-term child [tiab] OR Premature children [tiab] OR Pre-mature children [tiab] OR Preterm children [tiab] OR Pre-term children [tiab] OR Premature infant * [tiab] OR Pre-mature infant * [tiab] OR Preterm infant * [tiab] OR Pre-term infant * [tiab] OR Premature newborn * [tiab] OR Pre-mature newborn * [tiab] OR Preterm newborn * [tiab] OR Pre-term newborn * [tiab] OR Premature neonate * [tiab] OR Pre-mature neonate * [tiab] OR Preterm neonate * [tiab] OR Pre-term neonate * [tiab] OR Premature birth * [tiab] OR Pre-mature birth * [tiab] OR Preterm birth * [tiab] OR Pre-term birth * [tiab] OR Neonatal prematurity [tiab] OR prematuritas [tiab] OR “Hypertension, Pregnancy-Induced” [Mesh] OR Pre-Eclampsia [tiab] OR Preeclampsia [tiab] OR pre-eclamptic [tiab] OR preeclamptic [tiab] OR preclampsia [tiab] OR Proteinuria Edema Hypertension Gestosis [tiab] OR Edema Proteinuria Hypertension Gestosis [tiab] OR EPH Gestosis [tiab] OR EPH Toxemia * [tiab] OR EPH Complex [tiab] OR “Placental Insufficiency” [Mesh] OR “placenta insufficiency” [tiab] OR “placental insufficiency” [tiab] OR “placenta insufficiencies” [tiab] OR “placental insufficiencies” [tiab] OR “placenta deficiency” [tiab] OR “placental deficiency” [tiab] OR “placenta deficiencies” [tiab] OR “placental deficiencies” [tiab] OR “placenta failure” [tiab] OR “placental failure” [tiab] OR “Fetal Macrosomia” [Mesh] OR Macrosomia * [tiab] OR high birth weight [tiab] OR “overweight infant” [tiab] OR “overweight infants” [tiab] OR “overweight newborn” [tiab] OR “overweight newborns” [tiab] OR “overweight neonate” [tiab] OR “overweight neonates” [tiab] OR “Diabetes, Gestational” [Mesh] OR “Pregnancy in Diabetics” [Mesh] OR diabetes gravidarum [tiab]

Fourteen amino acids during pregnancy: arginine, citrulline, glutamate, glutamine, asparagine, aspartic acid, proline, ornithine, (iso-)leucine, valine, cysteine, methionine, and choline. The search was performed on 25 July 2018, with 14,169 records.

**Table A2:** Search terms in Embase.

14 amino acids AND administration	(exp Arginine/OR (Arginine OR L-Arginine).ti,ab.) OR (exp Leucine/OR exp Isoleucine/OR (Leucine OR L-Leucine OR Leucin OR Isoleucine OR Isoleucin OR Alloisoleucine OR Alloisoleucin).ti,ab.) OR (exp Valine/OR (Valine OR L-Valine OR Valsartan OR Valerate).ti,ab.) OR (exp Cysteine/OR (Cysteine OR L-Cysteine OR Cysteinate OR Acetylcysteine OR Carbocysteine OR Cysteinyldopa OR Cystine OR cystein OR cysthion OR Selenocysteine).ti,ab,kw.) OR (exp Methionine/OR (Methionine OR L-Methionine OR Liquimeth OR Pedameth OR Formylmethionine OR Racemethionine OR Adenosylmethionine OR Selenomethionine OR Vitamin U OR acimetion OR cotameth OR lobamine OR menin OR menine OR meonine OR methiolate OR methionin OR methiotrans OR methnine OR methurine OR metione OR methidin OR neutrodor OR oradash OR urosamine OR Methyl Donor).ti,ab,kw.) OR (exp glutamic acid/OR (glutamic acid OR glutamate OR MSG OR vestin OR aminoglutaric acid OR aminopentanedioic acid OR acidogen OR acidoride OR acidothym OR acidulin OR aciglumin OR aciglut OR aclor OR antalka OR flanithin OR gastuloric OR glusate OR glutadox OR glutamidin OR glutamin acid OR glutaminic acid OR glutaminol OR glutan OR glutansin OR glutasin OR glutaton OR hydrionic OR hypochylin OR levoglutamate OR levoglutamic acid OR muriamic OR pepsdol).ti,ab,kw.) OR (exp glutamine/OR (glutamine OR aminoglutaramic acid OR acutil OR adamin G OR glumin OR glutamin OR levoglutamide OR levoglutamine OR nutrestore).ti,ab,kw.) OR (exp Citrulline/OR (citrulline OR ureidopentanoic acid OR ureidonorvaline OR ureidovaleric acid OR citrullin OR carbamylornithine).ti,ab,kw.) OR (exp asparagine/OR (asparagine OR asparagin OR aminosuccinamic acid).ti,ab,kw.) OR (exp aspartic acid/OR (aspartic acid OR aspartate OR Magnesiocard OR Mg-5-Longoral OR Mg 5 Longoral OR Mg5 Longoral OR panangin OR astra 2045 OR aminosuccinic acid OR asparagic acid OR asparaginate OR asparaginic acid OR aspartyl OR aspatofort OR levoaspartic acid).ti,ab,kw.) OR (exp Proline/OR (proline OR prolin OR levoproline OR pyrrolidinecarboxylic acid OR pyrrolidine carboxylate).ti,ab,kw.) OR (exp ornithine/OR (ornithine OR ornithin OR Diaminopentanoic Acid OR diaminovaleric acid).ti,ab,kw.) OR (exp Choline/OR (choline OR bursine OR vidine OR fagine OR trimethylammonium hydroxide OR amonita OR bilineurine OR biocholine OR biocolina OR cholin OR hepacholine OR laevocholine OR levocholine OR lipotril OR luridine OR sincaline OR urocholine).ti,ab,kw.) AND (exp dietary supplement/OR exp nutrition supplement/OR (administration * OR administer * OR dose OR doses OR dosage OR treatment OR treated OR supplement * OR diet OR diets OR dietary OR intake OR intakes OR consumption OR consumptions OR consume OR nutraceutical * OR nutriceutical * OR therapy OR therapies).ti,ab,kw.)
AND	AND
Healthy pregnancy OR Complicated pregnancy	(exp pregnancy/OR exp fetus/OR (Pregnancy OR Pregnancies OR Pregnant OR Gestation OR Gestations OR Gestational OR gravidity OR gravidities OR gravid OR fetus OR foetus OR fetal OR foetal OR childbearing OR child bearing).ti,ab,kw.) OR (exp intrauterine growth retardation/OR exp Low Birth Weight/OR exp prematurity/OR (FGR OR Intrauterine growth retardation OR Intra-uterine growth retardation OR Intrauterine growth restriction OR Intra-uterine growth restriction OR IUGR OR Small for Gestational Age OR SGA OR Low birth weight OR Premature baby OR Pre-mature baby OR Preterm baby OR Pre-term baby OR Premature babies OR Pre-mature babies OR Preterm babies OR Pre-term babies OR Premature child OR Pre-mature child OR Preterm child OR Pre-term child OR Premature children OR Pre-mature children OR Preterm children OR Pre-term children OR Premature infant OR Premature infants OR Pre-mature infant OR Pre-mature infants OR Preterm infant OR Preterm infants OR Pre-term infant OR Pre-term infants OR Premature newborn OR Premature newborns OR Pre-mature newborn OR Pre-mature newborns OR Preterm newborn OR Preterm newborns OR Pre-term newborn OR Pre-term newborns OR Premature neonate OR Premature neonates OR Pre-mature neonate OR Pre-mature neonates OR Preterm neonate OR Preterm neonates OR Pre-term neonate OR Pre-term neonates OR Premature birth OR Premature births OR Pre-mature birth OR Pre-mature births OR Preterm birth OR Preterm births OR Pre-term birth OR Pre-term births OR Neonatal prematurity OR prematuritas).ti,ab,kw.) OR (exp maternal hypertension/OR (Pre-Eclampsia OR Preeclampsia OR pre-eclamptic OR preeclamptic OR preclampsia OR Proteinuria Edema Hypertension Gestosis OR Edema Proteinuria Hypertension Gestosis OR EPH Gestosis OR EPH Toxemia * OR EPH Complex).ti,ab,kw.) OR (exp Placenta Insufficiency/OR (placenta insufficiency OR placental insufficiency OR placenta insufficiencies OR placental insufficiencies OR placenta deficiency OR placental deficiency OR placenta deficiencies OR placental deficiencies OR placenta failure OR placental failure).ti,ab,kw.) OR (exp Macrosomia/OR (Macrosomia * OR high birth weight OR overweight infant OR overweight infants OR overweight newborn OR overweight newborns OR overweight neonate OR overweight neonates).ti,ab,kw.) OR (exp pregnancy diabetes mellitus/OR diabetes gravidarum.ti,ab,kw)

Fourteen amino acids during pregnancy: arginine, citrulline, glutamate, glutamine, asparagine, aspartate, proline, ornithine, (iso-)leucine, valine, cysteine, methionine, and choline. The search was performed on 25 July 2018, with 10,393 records.

**Table A3:** Search terms in Cochrane.

14 amino acids AND administration	(MeSH descriptor: [Glutamine] explode all trees and with qualifier(s): [Administration & dosage-AD, Adverse effects-AE, Deficiency-DF, Drug effects-DE, Pharmacology-PD, Physiology-PH, Therapeutic use-TU] OR MeSH descriptor: [Glutamic Acid] explode all trees OR MeSH descriptor: [Citrulline] explode all trees and with qualifier(s): [Administration & dosage—AD, Adverse effects-AE, Deficiency—DF, Drug effects—DE, Pharmacology—PD, Physiology—PH, Therapeutic use—TU] OR MeSH descriptor: [Asparagine] explode all trees and with qualifier(s): [Administration & dosage—AD, Adverse effects—AE, Deficiency—DF, Drug effects—DE, Pharmacology—PD, Physiology—PH, Therapeutic use—TU] OR MeSH descriptor: [Aspartic Acid] explode all trees and with qualifier(s): [Administration & dosage—AD, Adverse effects—AE, Deficiency—DF, Drug effects—DE, Pharmacology—PD, Physiology—PH, Therapeutic use—TU] OR MeSH descriptor: [Proline] explode all trees and with qualifier(s): [Administration & dosage—AD, Adverse effects—AE, Deficiency—DF, Drug effects—DE, Pharmacology—PD, Physiology—PH, Therapeutic use—TU] OR MeSH descriptor: [Ornithine] explode all trees and with qualifier(s): [Administration & dosage—AD, Adverse effects—AE, Deficiency—DF, Drug effects—DE, Pharmacology—PD, Physiology—PH, Therapeutic use—TU] OR MeSH descriptor: [Choline] explode all trees and with qualifier(s): Administration & dosage—AD, Adverse effects—AE, Pharmacology—PD, Physiology—PH, Therapeutic use—TU]MeSH descriptor: [Arginine] explode all trees and with qualifier(s): [Administration & dosage—AD, Adverse effects—AE, Deficiency—DF, Pharmacology—PD, Physiology—PH, Therapeutic use—TU] OR MeSH descriptor: [Leucine] explode all trees and with qualifier(s): [Administration & dosage—AD, Adverse effects—AE, Deficiency—DF, Pharmacology—PD, Physiology—PH, Therapeutic use—TU] OR MeSH descriptor: [Isoleucine] explode all trees and with qualifier(s): [Administration & dosage—AD, Adverse effects—AE, Deficiency—DF, Pharmacology—PD, Physiology—PH, Therapeutic use—TU] OR MeSH descriptor: [Valine] explode all trees and with qualifier(s): [Administration & dosage—AD, Adverse effects—AE, Deficiency—DF, Pharmacology—PD, Physiology—PH, Therapeutic use—TU] OR MeSH descriptor: [Cysteine] explode all trees and with qualifier(s): [Administration & dosage—AD, Adverse effects—AE, Deficiency—DF, Pharmacology—PD, Physiology—PH, Therapeutic use—TU] OR MeSH descriptor: [Methionine] explode all trees and with qualifier(s): [Administration & dosage—AD, Adverse effects—AE, Deficiency—DF, Pharmacology—PD, Physiology—PH, Therapeutic use—TU]) OR ((MeSH descriptor: [Dietary Supplements] explode all trees OR MeSH descriptor: [Administration, Oral] explode all trees OR administration * OR administer * OR dose OR doses OR dosage OR treatment OR treated OR supplement * OR diet OR diets OR dietary OR intake OR intakes OR consumption OR consumptions OR consume OR nutraceutical * OR nutriceutical * OR therapy OR therapies:ti,ab,kw ) AND (MeSH descriptor: [Glutamic Acid] explode all trees OR MeSH descriptor: [Glutamine] this term only OR MeSH descriptor: [Citrulline] explode all trees OR MeSH descriptor: [Asparagine] explode all trees OR MeSH descriptor: [Aspartic Acid] this term only OR MeSH descriptor: [D-Aspartic Acid] explode all trees OR MeSH descriptor: [Proline] this term only OR MeSH descriptor: [Choline] this term only OR MeSH descriptor: [Ornithine] this term only OR glutamic acid OR glutamate OR MSG OR vestin OR “aminoglutaric acid” OR “aminopentanedioic acid” OR acidogen or acidoride OR acidothym OR acidulin OR aciglumin OR aciglut OR aclor OR antalka OR flanithin OR gastuloric OR glusate OR glutadox OR glutamidin OR “glutamin acid” OR “glutaminic acid” OR glutaminol OR glutan OR glutansin OR glutasin OR glutaton OR hydrionic OR hypochylin OR levoglutamate OR “levoglutamic acid” OR muriamic OR pepsdol or glutamine OR “aminoglutaramic acid” or acutil OR “adamin G” OR glumin OR glutamin OR levoglutamide OR levoglutamine OR nutrestore or citrulline OR “ureidopentanoic acid” OR ureidonorvaline OR “ureidovaleric acid” OR citrullin OR carbamylornithine OR asparagine OR asparagin OR “aminosuccinamic acid” OR “aspartic acid” OR aspartate OR Magnesiocard OR Mg-5-Longoral OR Mg 5 Longoral OR Mg5 Longoral OR panangin OR astra 2045 OR “aminosuccinic acid” OR “asparagic acid” OR asparaginate OR “asparaginic acid” OR aspartyl OR aspatofort OR “levoaspartic acid” OR proline OR prolin OR levoproline OR “pyrrolidinecarboxylic acid”OR pyrrolidine carboxylate OR ornithine OR ornithin OR “Diaminopentanoic Acid” OR “diaminovaleric acid” OR choline OR bursine OR vidine OR fagine OR trimethylammonium hydroxide OR amonita OR bilineurine OR biocholine OR biocolina OR cholin OR hepacholine OR laevocholine OR levocholine OR lipotril OR luridine OR sincaline OR urocholine:ti,ab,kw OR MeSH descriptor: [Arginine] explode all trees OR MeSH descriptor: [Leucine] explode all trees OR MeSH descriptor: [Isoleucine] explode all trees OR MeSH descriptor: [Valine] explode all trees OR MeSH descriptor: [Cysteine] explode all trees OR MeSH descriptor: [Methionine] explode all trees OR Arginine OR L-Arginine OR Leucine OR L-Leucine OR Leucin OR Isoleucine OR Isoleucin OR Alloisoleucine OR Alloisoleucin OR Valine or L-Valine OR Valsartan OR Valerate OR Cysteine OR L-Cysteine OR Cysteinate OR Acetylcysteine OR Carbocysteine OR Cysteinyldopa OR Cystine OR cystein OR cysthion OR Selenocysteine OR Methionine OR L-Methionine OR Liquimeth OR Pedameth OR formylmethionine OR Racemethionine OR Adenosylmethionine OR Selenomethionine OR Vitamin U OR acimetion OR cotameth OR lobamine OR menin OR menine OR meonine OR methiolate OR methionin OR methiotrans OR methnine OR methurine OR metione OR methidin OR neutrodor OR oradash OR urosamine OR Methyl Donor:ti,ab,kw))
AND	AND
Healthy pregnancy OR Complicated pregnancy	MeSH descriptor: [Pregnancy] explode all trees OR MeSH descriptor: [Gravidity] explode all trees OR MeSH descriptor: [Fetus] explode all trees OR MeSH descriptor: [Infant, Low Birth Weight] explode all trees OR MeSH descriptor: [Infant, Premature] explode all trees OR MeSH descriptor: [Premature Birth] explode all trees OR MeSH descriptor: [Hypertension, Pregnancy-Induced] explode all trees OR MeSH descriptor: [Fetal Macrosomia] explode all trees OR MeSH descriptor: [Diabetes, Gestational] explode all trees OR MeSH descriptor: [Pregnancy in Diabetics] explode all trees OR MeSH descriptor: [Placental Insufficiency] explode all trees OR Pregnancy OR Pregnancies OR Pregnant OR Gestation OR Gestations OR Gestational OR gravidity OR gravidities OR gravid or fetus OR foetus OR fetal OR foetal OR childbearing OR “child bearing” OR FGR OR Intrauterine growth retardation OR Intra-uterine growth retardation OR Intrauterine growth restriction OR Intra-uterine growth restriction OR IUGR OR Small for Gestational Age OR SGA OR Low birth weight OR Premature baby OR Pre-mature baby OR Preterm baby OR Pre-term baby OR Premature babies OR Pre-mature babies OR Preterm babies OR Pre-term babies OR Premature child OR Pre-mature child OR Preterm child OR Pre-term child OR Premature children OR Pre-mature children or Preterm children OR Pre-term children OR Premature infant * OR Pre-mature infant * OR Preterm infant * OR Pre-term infant * OR Premature newborn * OR Pre-mature newborn * OR Preterm newborn * OR Pre-term newborn * OR Premature neonate * OR Pre-mature neonate * OR Preterm neonate * OR Pre-term neonate * OR Premature birth * OR Pre-mature birth * PR Preterm birth * OR Pre-term birth * OR Neonatal prematurity OR prematuritas OR Pre-Eclampsia OR Preeclampsia OR pre-eclamptic OR preeclamptic OR preclampsia OR Proteinuria Edema Hypertension Gestosis OR Edema Proteinuria Hypertension Gestosis OR EPH Gestosis or EPH Toxemia * OR EPH Complex OR “placenta insufficiency” OR “placental insufficiency” OR “placenta insufficiencies” OR “placental insufficiencies” OR “placenta deficiency” OR “placental deficiency” OR “placenta deficiencies” OR “placental deficiencies” OR “placenta failure” OR “placental failure” OR Macrosomia * OR high birth weight OR “overweight infant” OR “overweight infants” OR “overweight newborn” OR “overweight newborns” OR “overweight neonate” OR “overweight neonates” OR diabetes gravidarum:ti,ab,kw

Fourteen amino acids during pregnancy: arginine, citrulline, glutamate, glutamine, asparagine, aspartate, proline, ornithine, (iso-)leucine, valine, cysteine, methionine, and choline. The search was performed on 25 July 2018, with 819 records.

**Table A4:** Main study characteristics and reported outcome of included studies.

Author (Year)	Species	Animal Model	Pregnancy Complication	Dose (g/kg/day)	Fetal/Birth Weight	Maternal BP	Glucose	GWG	Dev. Compl
**Arginine**									
Greene (2012) [33]	Mouse	Control	Normal	2.664 ^a^	X			X	
Alexander (2004) [34]	Rat	RUPP	FGR/PE	2.000 ^a^	X	X			
		Control	Normal	2.000 ^a^	X	X			
Altun (2008) [35]	Rat	Sonic stress-induction	PE	0.021	X	X			
Bourdon (2016) [44]	Rat	Protein restriction	FGR	1.500	X			X	
Bursztyn (2003) [45]	Rat	Hyperinsulinemia	FGR/PIH	0.217	X	X	X	X	
Chin (1991) [46]	Rat	Control	Normal	0.159	X			X	
Da Costa (2014) [47]	Rat	Control	Normal	0.021	X				
Helmbrecht (1996) [48]	Rat	L-NAME	FGR/PE	0.021	X	X			
		Control	Normal	0.021	X	X			
Podjarny (1993) [90]	Rat	Control	Normal	0.322		X		X	
		Adriamycin nephropathy	PE	0.322		X		X	
Podjarny (1997) [92]	Rat	Adriamycin nephropathy	PE	0.230		X			
Podjarny (2001a) [49]	Rat	Hyperinsulinemia	PIH	0.220	X	X	X	X	
Podjarny (2001b) [91]	Rat	Control	Normal	0.260		X		X	
		L-NAME	FGR/PE	0.260		X		X	
Schooley (2002) [50]	Rat	Mg deficiency	FGR	1.000	X	X			
		Control	No	1.000	X	X			
Sharkey (2001) [51]	Rat	Genetic	FGR/PIH	0.100 ^a,b^	X	X			
Vosatka (1998) [36]	Rat	Hypobaric hypoxia	FGR	0.200 ^a^	X				
				2.000 ^a^	X			X	
Zeng (2008) [37]	Rat	Control	Normal	0.216	X			X	
					X			X	
Crane (2016) [52]	Sheep	Control	Normal	0.150	X				
Sun (2017) [54]	Sheep	Diet restriction twin bearing	FGR	0.250	X			X	
Sun (2018) [55]	Sheep	Diet restriction twin bearing	FGR	0.249	X			X	
Zhang (2016a) [56]	Sheep	Diet restriction twin bearing	FGR	0.250	X				
Zhang (2016b) [57]	Sheep	Diet restriction twin bearing	FGR	0.250	X		X	X	
Peine (2018) [53]	Sheep	Diet restriction	FGR	0.180	X			X	
Bass (2017) [58]	Pig	Control	Normal	0.150	X			X	
				0.170	X			X	
Bérard (2010) [59]	Pig	Control	Normal	0.160	X			X	
Che (2013) [70]	Pig	Control	Normal	0.082 ^a^	X				
Dallanora (2017) [69]	Pig	Control	Normal	0.115	X			X	
Gao (2012) [71]	Pig	Control	Normal	0.140 ^a^	X				
Garbossa (2015) [72]	Pig	Control	Normal	0.113 ^a^	X				
		Ractopamine	Over-growth	0.113 ^a^	X				
Guo (2016) [73]	Pig	Control	Normal	0.015 ^a^	X				
Li (2010) [74]	Pig	Control	Normal	0.070	X		X	X	
				0.140	X		X	X	
Li (2014) [75]	Pig	Control	Normal	0.070	X			X	
				0.140	X			X	
Li (2015) [76]	Pig	Control	Normal	0.210	X			X	
				0.140	X			X	
				0.130	X			X	
Liu (2012) [77]	Pig	Control	Normal	0.107	X		X		
Madsen (2017) [60]	Pig	UOL	Normal	0.157 ^a^	X				
		Intact (relatively crowded)	FGR	0.157 ^a^	X				
Mateo (2007) [61]	Pig	Control	Normal	0.120	X				
Mateo (2008) [62]	Pig	Control	Normal	0.120	X				
Wu (2012) [63]	Pig	Control	Normal	0.110	X				
Quesnel (2014) [64]	Pig	Control	Normal	0.110	X				
**Camarena Pulido** (2016) [78]	Human	Increased risk of PE	Risk	0.042 ^a^	X	X			X
Dera (2007) [95]	Human	FGR < p10; gestational HTN	Risk	0.043 ^a^				X	X
Facchinetti (2007) [79]	Human	HTN after 20th wk	Risk	0.110 ^a^	X				X
Hladenuwich (2006) [82]	Human	PE	PE	0.200 ^a^	X				
Neri (2010) [83]	Human	Chronic HTN	Risk	0.047 ^a^	X	X			X
Ropacka (2007) [84]	Human	EFW < p10	FGR	0.043 ^a^	X				
Rytlewski (2005) [93]	Human	PE; BW < p10	FGR/PE	0.040		X			
Rytlewski (2006) [85]	Human	PE; BW < p10	FGR/PE	0.041	X				X
Rytlewski (2008) [86]	Human	Preterm labor sign	Preterm	0.041 ^a^	X				X
Sieroszewski (2004) [87]	Human	EFW < p10	FGR	0.040 ^a^	X				X
Singh (2015) [88]	Human	EFW < p10	FGR	0.065 ^a^	X				
Staff (2004) [89]	Human	PE	FGR/PE	0.150 ^a^	X	X			
Valdivia-silva (2009) [96]	Human	PE	PE	0.039					X
Winer (2009) [80]	Human	AC < p3 and abnormal UA flow	FGR	0.190	X	X			
Zhang (2007) [81]	Human	PIH and FGR	FGR/PIH	0.333	X				
**Citrulline**									
Bourdon (2016) [44]	Rat	Protein restriction	FGR	2.000	X	X		X	
Koeners (2017) [38]	Rat	Pre-existent HTN	PIH	0.438 ^b^	X				
Tran (2017) [39]	Rat	Protein restriction	FGR	2.000	X				
Powers (2015) [94]	Human	Obese	Normal	0.035 ^a^		X			
**Glutamate**									
Beaudoin (1981) [40]	Rat	Control	Normal	2.600 ^a^	X				
Navarro (2005) [148]	Rat	Control	Normal	0.110				X	
Zeng (2012) [41]	Rat	Control	Normal	0.074 ^b^	X				
				0.149 ^b^	X				
Sun (2017) [54]	Sheep	Diet restriction twin bearing	FGR	0.060	X			X	
Sun (2018) [55]	Sheep	Diet restriction twin bearing	FGR	0.062	X			X	
Zhang (2016a) [56]	Sheep	Diet restriction twin bearing	FGR	0.060	X				
Zhang (2016b) [57]	Sheep	Diet restriction twin bearing	FGR	0.060	X		X		
Liu (2012) [77]	Pig	Control	Normal	0.011	X		X		
Wu (2012) [63]	Pig	Control	Normal	0.011	X				
Zhang (2014) [65]	Pig	Control	Normal	0.007	X				
				0.015	X				
				0.022	X				
				0.030	X				
Zhu (2015) [149]	Pig	Diet restriction twin bearing	FGR	0.060				X	
Cai (2018) [66]	Swine	Control	Normal	0.003	X			X	
					X			X	
					X			X	
**Glutamine**									
Caporossi (2014) [42]	Rat	Control	Normal	2.000	X				
Zhu (2018) [67]	Pig	Control	Normal	0.126	X		X	X	
**Proline**									
Gonzalez-Anover (2017) [68]	Pig	Control	Normal	0.120 ^a^	X				
**Aspartic acid**									
Brunner (1978) [43]	Rat	PKU-induction	FGR	1.570 ^b^	X			X	
		Control	Normal	1.570 ^b^	X			X	
**Valine**									
Brunner (1978) [43]	Rat	PKU-induction	FGR	1.570 ^b^	X			X	
				4.720 ^b^	X			X	
		Control	Normal	1.570 ^b^	X			X	
				4.720 ^b^	X			X	
Matsueda (1982) [97]	Rat	Control	Normal	2.710	X			X	
Mori (1999) [98]	Rat	Control	Normal	2.368	X			X	
Xu (2017) [104]	Pig	Control	Normal	0.037	X				
				0.087	X				
**Leucine**									
Viana (2013) [103]	Mouse	Control	Normal	3.000 ^a,b^	X				
Brunner (1978) [43]	Rat	PKU-induction	FGR	1.570 ^b^	X			X	
				4.720 ^b^	X			X	
		Control	Normal	1.570 ^b^	X			X	
				4.720 ^b^	X			X	
Cruz (2014) [99]	Rat	Control	Normal	NA	X				
Cruz (2016) [100]	Rat	Control	Normal	NA	X				
Matsueda (1982) [97]	Rat	Control	Normal	1.500	X			X	
Mawatari (2004) [102]	Rat	Control	Normal	0.300	X			X	
		Control	Normal	1.000	X			X	
Mori (1999) [98]	Rat	Control	Normal	2.368	X			X	
Ventrucci (2001) [101]	Rat	Control	Normal	7.180 ^b^	X			X	
Ventrucci (2002) [150]	Rat	Control	Normal	7.180 ^b^			X		
Wang (2018) [105]	Pig	Control	Normal	0.046	X				
				0.092	X				
				0.138	X				
**Isoleucine**									
Brunner (1978) [43]	Rat	PKU-induction	FGR	1.570 ^b^	X			X	
				4.720 ^b^	X			X	
		Control	Normal	1.570 ^b^	X			X	
				4.720 ^b^	X			X	
Matsueda (1982) [97]	Rat	Control	Normal	3.150	X			X	
Mori (1999) [98]	Rat	Control	Normal	2.368	X			X	
**Cysteine**									
Balansky (2009) [106]	Mouse	Control	Normal	1.000	X				
Miller (2014) [108]	Mouse	Control	Normal	0.150	X			X	
Moazzen (2014) [107]	Mouse	Streptozotocin	Pre-existent DM	1.000	X		X		
		Control	Normal	1.000	X		X		
Lin (2011) [113]	Rat	High-fat diet	Over-growth	0.075 ^a,b^	X		X		
Soto-Blanco (2001) [114]	Rat	Control	Normal	0.510	X			X	
Hashimoto (2012) [120]	Guinea pig	Hypoxia	FGR	0.550	X				
		Normoxia	Normal	0.550	X				
Herrera (2017) [121]	Guinea pig	Control	Normal	0.500	X				
		Uterine artery occlusion	FGR	0.500	X				
Thompson (2011) [122]	Guinea pig	Nicotine exposed	Risk	0.496	X				
Motawei (2016) [133]	Human	PE	PE	0.005	X	X			
Roes (2006) [134]	Human	Early onset severe PE/HELLP	PE	0.025 ^a^	X				X
Shahin (2009) [137]	Human	Previous preterm labor	Risk	0.008 ^a^	X				X
**Methionine**									
Abdel-Wanhab (1999) [115]	Rat	Control	Normal	0.043	X				
Brunner (1978) [43]	Rat	PKU-induction	FGR	1.570 ^b^	X			X	
		Control	Normal	1.570 ^b^	X			X	
Chandrashekar (1977) [116]	Rat	Control	Normal	NA	X				
Fujii (1971) [117]	Rat	Control	Normal	0.029	X				
Koz (2010) [118]	Rat	Control	Normal	1.000	X				
Matsueda (1982) [97]	Rat	Control	Normal	1.150	X			X	
Viau (1973) [119]	Rat	Control	Normal	NA	X				
Gauthier (2009) [123]	Guinea pig	Ethanol exposed	FGR	0.096	X				
Othmani Mecif (2017) [124]	Rabbit	Control	Normal	0.172	X				
Liu (2016) [125]	Sheep	Control	Normal	0.047	X			X	
Tsiplakou (2017) [126]	Sheep	Control	Normal	0.041	X				
Mori (1999) [98]	Rat	Control	Normal	2.368	X			X	
Batistel (2017) [128]	Cow	Control	Normal	0.012 ^a^	X				
Clements (2017) [129]	Cow	Control	Normal	0.016	X				
Jacometo (2016) [130]	Cow	Control	Normal	0.010 ^b^	X				
Xu (2018) [131]	Cow	Control	Normal	0.012 ^a,b^	X				
**Choline**									
Bai (2012) [109]	Rat	Protein restriction	FGR	0.670 ^a^	X			X	
Thomas (2009) [110]	Rat	Ethanol exposed	FGR	0.250	X				
		Control	Normal	0.250	X				
Yang (2000) [111]	Rat	Control	Normal	0.390 ^a,b^	X				
Zhang (2018) [112]	Rat	LPS infusion	FGR	0.875 ^b^	X	X		X	
		Control	Normal	0.875 ^b^	X	X		X	
Birch (2016) [127]	Sheep	Ethanol infusion	FGR	0.010	X				
		Control	Normal	0.010	X				
Janovick Guretzky (2006) [151]	Cow	Control (Holstein)	Normal	0.021				X	
		Control (Jersey)	Normal	0.030				X	
Zenobi (2018a) [132]	Cow	Excess energy diet	Risk overgrowth	0.024	X		X		
		Maintenance diet	Normal	0.024	X		X		
Zenobi (2018b) [152]	Cow	Diet restriction	FGR	0.002			X		
				0.003			X		
				0.005			X		
				0.007			X		
		Ad libitum diet	Normal	0.002			X		
				0.003			X		
				0.005			X		
				0.007			X		
Jacobson (2018) [138]	Human	Alcohol	Risk	0.035	X	X		X	X
Ross (2013) [135]	Human	Control	Normal	0.012 ^a^	X			X	X
Yan (2012) [136]	Human	Control	Normal	0.007 ^a^	X			X	

Ordered according to species per amino acid. ^a^ Dose in g/kg/day was calculated using the estimated mean maternal weight or based on ^b^ estimated food intake. “Normal” in the pregnancy complication column indicates the normal-growth group. AA, amino acid; BP, blood pressure; BW, birth weight; dev. compl; development of pregnancy complication in risk population; DM, diabetes mellitus; EFW, estimated fetal weight; FGR, fetal growth restriction; GWG, gestational weight gain; L-NAME, (ω)-nitro-L-arginine methyl ester; LPS, lipopolysaccharides; NA: not applicable or available; HELLP, hemolysis, elevated liver enzyme, and low platelet syndrome; HTN, hypertension; PE, preeclampsia; PKU; phenylketonuria; PIH, pregnancy-induced hypertension; RUPP, reduced uterine pressure perfusion; UA, uterine artery; UOL, unilateral oviduct ligation; WT, wild type.

**Table A5:** Data-extraction of included animal and human studies on birth weight.

Author (Year)	Species (Strain or Coumtry)	Animal Model	Pregnancy Complication	Maternal Weight (kg)	Dietary Protein Intake (%)	Supplementation Scheme ^a^; intervention Type ^b^	Supplementation Duration (GD)	Daily Dose (g/kg Body wt)	Measurement Day (GD)	AA BW ± SD (g)	*n* Offspring (Sex ^c^)	*n* Mother	CON BW ± SD (g)	*n* Offspring (Sex ^c^)	*n* Mother
**Arginine**															
Greene (2012) [33]	Mouse (FNVB/N × vegfr2-luc)	Control	Normal	0.03	24	C; T ^d^	1–18	2.660 ^e^	P0	1.18 ± 0.28	49	6	1.32 ± 0.25	25	5
Alexander (2004) [34]	Rat (SD)	RUPP	FGR/PE	0.25	NA	C; P	10–19	2.000 ^e^	19	2.30 ± 0.60	117	9	2.00 ± 0.60	117	9
		Control	Normal	0.25	NA	C; P	10–19	2.000 ^e^	19	2.80 ± 0.60	117	9	2.40 ± 0.35	156	12
Altun (2008) [35]	Rat (Wistar)	Sonic stress–induction	PE	0.20	NA	C; T	18–19	0.021	19	5.18 ± 0.58	67	6	4.41 ± 0.46	55	6
Bourdon (2016) [44]	Rat (SD)	Protein restriction	FGR	0.29	4	C; T ^d^	7–21	1.500	21	4.13 ± 0.30	111	9	4.05 ± 0.15	110	9
Bursztyn (2003) [45]	Rat (Wistar)	Hyperinsulinemia	FGR/PIH	0.24	NA	C; T	11–22	0.217	22	4.10 ± 0.20	65	5	3.00 ± 1.60	55	5
Chin (1991) [46]	Rat (Fisher F344/NTacfBR)	Control	Normal	0.17	NA	C; P ^d^	1-P0	0.159	P0	4.60 ± 1.88	40	4	4.90 ± 1.88	28	4
Da Costa (2014) [47]	Rat (Wistar)	Control	Normal	0.20	NA	I; P	4–18	0.021	18	469.33 ± 124.55	NA	5	368.00 ± 138.12	NA	5
Helmbrecht (1996) [48]	Rat (SD)	L–NAME	FGR/PE	0.31	NA	C; T	16–21	0.021	P0	5.60 ± 0.32	126	6	5.00 ± 0.17	120	6
		Control	Normal	0.31	NA	C; P	16–21	0.021	P0	6.11 ± 0.37	78	6	6.00 ± 0.27	80	6
Podjarny (2001a) [49]	Rat (Wistar)	Hyperinsulinaemia	PIH	0.24	NA	C; P	11–22	0.220	22	5.60 ± 0.39	180	15	4.30 ± 1.34	200	20
Schooley (2002) [50]	Rat (SD)	Mg deficiency	FGR	0.20	NA	C; T	10–21	1.000	21	3.97 ± 0.40	146	13	3.64 ± 0.42	133	12
		Control	Normal	0.20	NA	C; P	10–21	1.000	21	4.10 ± 0.40	134	13	3.80 ± 0.45	129	14
Sharkey (2001) [51]	Rat (SHHF/Mcc-facp)	Genetic	FGR/PIH	0.20	NA	C; P	1–20	0.100 ^e,f^	20	1.57 ± 0.07	NA	3	2.68 ± 0.12	NA	4
Vosatka (1998) [36]	Rat (Wistar)	Hypobaric hypoxia	FGR	0.25	4 g/day	C; P	9–21	0.200 ^e^	21	4.19 ± 0.41	38	4	3.30 ± 0.65	137	14
								2.000 ^e^	21	4.27 ± 0.80	116	10	3.30 ± 0.65	137	14
Zeng (2008) [37]	Rat (SD)	Control	Normal	0.23	22	C; P ^d^	0-P0	0.216	P0	6.73 ± 0.87	174	12	6.43 ± 1.01	136	12
Crane (2016) [52]	Sheep (Rambouillet)	Control	Normal	65.0	16	C; P	0–14	0.150	P0	5300 ± 1073	20	20	5400 ± 1200	29	25
Peine (2018) [53]	Sheep (Rambouillet-cross)	Diet restriction	FGR	67.7	16	C; P	54-P0	0.180	P0	4603.00 ± 1454	11	11	4449 ± 1454	11	11
Sun (2017) [54]	Sheep (Hu)	Diet restricted twin bearing	FGR	40.1	NA	C; P	35–110	0.250	110	1660 ± 877	16	8	1400 ± 396	16	8
Sun (2018) [55]	Sheep (Hu)	Diet restricted twin bearing	FGR	40.1	10–14	C; T	35–110	0.249	110	1703 ± 154	16	8	1431 ± 156	16	8
Zhang (2016a) [56]	Sheep (Hu)	Diet restricted twin bearing	FGR	40.1	10–14	C; P	35–110	0.250	110	1660 ± 2998	16	8	1410 ± 2998	16	8
Zhang (2016b) [57]	Sheep (Hu)	Diet restricted twin bearing	FGR	40.1	10–14	C; P	35–110	0.250	110	1659 ± 130	16	8	1401.00 ± 130.11	16	8
Bass (2017) [58]	Pig (GPK-35)	Control	Normal	180	18.7	C; P ^d^	93-P0	0.150	P0	1420 ± 197	682	49	1410 ± 197	652	48
	Pig (PIC Camborough 1050 and 1055)	Control	Normal	165	12	C; P ^d^	81–116	0.170	P0	1410 ± 391	2515	195	1450 ± 391	2388	188
Bérard (2010) [59]	Pig (Swiss Large White)	Control	Normal	159	NA	C; P	14–28	0.160	75	373 ± 63	29 (F)	10	365 ± 63	20 (F)	7
Che (2013) [70]	Pig (Landrace × large White)	Control	Normal	236	14	C; P ^d^	30–90	0.082 ^e^	P0	13,900 ± 268	212	20	1480 ± 224	204	20
							30–114	0.092 ^e^	P0	1500 ± 224	236	20	1480 ± 224	204	20
Dallanora (2017) [69]	Pig (Landrace × Large white)	Control	Normal	148	17	C; P	25–112	0.115	80–112	1170 ± 282	755	51	1160 ± 282	796	51
Gao (2012) [71]	Pig (Yorkshire × Landrace)	Control	Normal	160	13	C; P ^d^	22-P0	0.140 ^e^	P0	1450 ± 624	642	52	1410 ± 624	630	56
Garbossa (2015) [72]	Pig	Control	Normal	160	14	C; P	25–53	0.113 ^e^	110	1430 ± 379	334	23	1380 ± 379	305	23
		Ractopamine	Overgrowth	160	14	C; T	25–53	0.113 ^e^	110	1510 ± 379	334	22	1500 ± 379	305	22
Guo (2016) [73]	Pig (Yorkshire × Landrace)	Control	Normal	150	16	C; P ^d^	30–110	0.015 ^e^	P0	1250 ± 319	60	60	1250 ± 319	53	53
Li (2010) [74]	Pig (Yorkshire × Landrace)	Control	Normal	112	12	C; P ^d^	0–25	0.070	25	8.90 ± 3.08	111	9	8.30 ± 4.08	114	9
								0.140	25	5.50 ± 4.08	77	8	8.30 ± 4.08	114	9
Li (2014) [75]	Pig (Yorkshire × Landrace)	Control	Normal	115	12	C; P ^d^	14–25	0.070	25	6.27 ± 1.18	191	15	5.58 ± 1.18	147	14
								0.140	25	5.83 ± 1.18	171	14	5.58 ± 1.18	147	14
Li (2015) [76]	Pig (Landrace)	Control	Normal	125	16	C; P ^d^	1–30	0.210	P0	1440 ± 340	354	32	1460 ± 397	306	30
				187	16	C; P ^d^	1–30	0.140	P0	1440 ± 397	756	57	1510 ± 397	651	56
				196	16	C; P ^d^	1–30	0.130	P0	1400 ± 374	481	37	1440 ± 374	123	37
Liu (2012) [77]	Pig (Landrace x Large White)	Control	Normal	187	15	C; P	1-P0	0.107	P0	1510 ± 104	98	9	1450 ± 104	88	9
Madsen (2017) [60]	Pig (Swiss Large White)	UOL	Normal	159	15	C; P ^d^	14–28	0.157 ^e^	P0	1440 ± 411	NA (F)	5	1500 ± 411	NA (F)	5
											NA (M)	5	1740 ± 353	NA (M)	5
		Intact (relatively crowded)	FGR	159	15	C; T ^d^	14–28	0.157 ^e^	P0	1510 ± 411	NA (F)	5	1260 ± 411	NA (F)	5
											NA (M)	5	1310 ± 353	NA (M)	5
Mateo (2007) [61]	Pig (Camborough 22)	Control	Normal	166	12	C; P ^d^	30-P0	0.120	P0	1460 ± 288	273	24	1410 ± 288	262	28
Mateo (2008) [62]	Pig (Camborough 22)	Control	Normal	166	12	C; P ^d^	30-P0	0.120	P0	1434 ± 154	NA	21	1430 ± 154		17
Wu (2012) [63]	Pig (Landrace × Large White)	Control	Normal	187	15	C; P	90-P0	0.110	P0	16,200 ± 104	97	9	1460 ± 104	83	9
Quesnel (2014) [64]	Pig (Landrace × Large White)	Control	Normal	236	13	C; P	70-P0	0.110	P0	1520 ± 376	357	24	1460 ± 379	317	23
Camarena Pulido (2016) [78]	Human (Mexico)	Increased risk of PE	Risk	71	NA	I; P	Wk 20-P0	0.042 ^e^	P0	3144 ± 454	49	49	2937 ± 491	47	47
Facchinetti (2007) [79]	Human (Italy)	HTN after 20th wk	Risk	80	NA	I; P	2 wks	0.110 ^e^	P0	2753 ± 857	39	39	2523 ± 803	35	35
Hladenuwich (2006) [82]	Human (USA)	PE	PE	70	NA	I; T	6 days	0.200 ^e^	P0	1734 ± 680	10	10	1653 ± 602	10	10
Neri (2010) [83]	Human (Italy)	Chronic HTN < 16 wk	Risk	85	NA	I; P	10–12 wks duration	0.047 ^e^	P0	3094 ± 719	39	39	2836 ± 946	40	40
Ropacka (2007) [84]	Human (Poland)	FGR (EFW < p10)	FGR	70	NA	I; T	Until P0 (±35 days)	0.043 ^e^	P0	2526 ± 844	24	24	1996 ± 928	17	17
Rytlewski (2006) [85]	Human (Poland)	PE; BW < p10	FGR/PE	74	NA	I; T	Wk 29-P0	0.041	P0	2358 ± 901	30	30	2066 ± 917	31	31
Rytlewski (2008) [86]	Human (Poland)	Preterm labor sign	Preterm	74	NA	I; T	1–4 wks after admission-delivery	0.041 ^e^	P0	2956 ± 538	25	25	2987 ± 474	20	20
Sieroszewski (2004) [87]	Human (Poland)	EFW < p10	FGR	75	NA	I; T	20 days (start wk 32)	0.040 ^e^	P0	2823 ± 751	78	78	2495 ± 805	30	30
Singh (2015) [88]	Human (India)	EFW < p10	FGR	48	NA	I; T	21 days	0.065 ^e^	P0	1900 ± 380	30	30	1770 ± 530	30	30
Staff (2004) [89]	Human (Norway)	PE	FGR/PE	80	NA	I; T	5 days	0.150 ^e^	P0	2264 ± 833	15	15	1986 ± 905	15	15
Winer (2009) [80]	Human (France)	AC < p3 and abnormal UA flow	FGR	75	NA	I; T	Until P0 (±3 wk)	0.190	P0	1042 ± 476	21	21	1068 ± 452	22	22
Zhang (2007) [81]	Human (China)	PIH and FGR	FGR/PIH	60	NA	I; T	Wk 28–30	0.333	P0	2900 ± 300	35	35	2700 ± 300	33	33
**Citrulline**															
Bourdon (2016) [44]	Rat (SD)	Protein restriction	FGR	0.29	4	C; T	7–21	2.000		4.15 ± 0.81	102	9	4.05 ± 0.15	110	9
Koeners (2007) [38]	Rat (SHR)	Pre-existent HTN	PIH	0.20	NA	C; T	7-P0	0.438 ^f^	P0	5.10 ± 0.56	14 (F)	7	4.88 ± 0.74	16 (F)	5
										5.32 ± 0.80	14 (M)	7	5.50 ± 0.52	17 (M)	5
Tran (2017) [39]	Rat (SD)	Protein restriction	FGR	0.20	4	C; P	2–15	2.000	15	0.29 ± 0.03	18	3	0.40 ± 0.02	24	3
							2–21	2.000	21	4.88 ± 0.42	30	3	4.62 ± 0.33	46	3
**Glutamate**															
Beaudoin (1981) [40]	Rat (Wistar Albino)	Control	Normal	0.23	NA	I; P	6–10	2.600 ^e^	20	3.96 ± 0.02	54	4	3.97 ± 0.04	57	5
Zeng (2012) [41]	Rat (SD)	Control	Normal	0.24	NA	C; P	1-P0	0.074 ^f^	P0	6.37 ± 0.29	1171	96	6.38 ± 0.29	1094	96
								0.149 ^f^	P0	6.36 ± 0.20	1248	96	6.38 ± 0.29	1094	96
Sun (2017) [54]	Sheep (Hu)	Diet restricted twin bearing	FGR	40.1	NA	C; P	35–110	0.060	220	1680 ± 1188	16	8	1400 ± 396	16	8
Sun (2018) [55]	Sheep (Hu)	Diet restricted twin bearing	FGR	40.1	10–14	C; T	35–110	0.062	110	1718 ± 110	16	8	1431 ± 156	16	8
Zhang (2016a) [56]	Sheep (Hu)	Diet restricted twin bearing	FGR	40.1	10–14	C; P	35–110	0.060	110	1680 ± 2998	16	8	1410 ± 2998	16	8
Zhang (2016b) [57]	Sheep (Hu)	Diet restricted twin bearing	FGR	40.1	10–14	C; P	35–110	0.060	110	1682 ± 130	16	8	1401 ± 130	16	8
Liu (2012) [77]	Pig (Landrace × Large White)	Control	Normal	187	15	I; P	1-P0	0.011	P0	1490 ± 104	97	9	1450 ± 104	88	9
Wu (2012) [63]	Pig (Landrace × Large White)	Control	Normal		NA	I; P	90-P0	0.011	P0	1590 ± 104	95	9	1460 ± 104	83	9
Zhang (2014) [65]	Pig (Landrace × Large White)	Control	Normal	136	15	C; P	1-P0	0.007	P0	1460 ± 67	99	9	1370 ± 67	89	9
								0.015	P0	1410 ± 67	97	9	1370 ± 67.08	89	9
								0.022	P0	1430 ± 67	98	9	1370.00 ± 67	89	9
								0.030	P0	1360 ± 67	95	9	1370 ± 67	89	9
Cai (2018) [66]	Swine (Landrace × Yorkshire)	Control	Normal	210	13	C; P	1–8	0.003	P0	1450 ± 415	177	18	1440 ± 415	173	18
							9–28	0.003	P0	1430 ± 415	166	16	1440 ± 415	173	18
							1–28	0.003	P0	1400 ± 415	207	17	1440 ± 415	173	18
**Glutamine**															
Caporossi (2014) [42]	Rat (Wistar)	Control	Normal	0.24	NA	I; P	1–21	2.000	21	3.53 ± 2.11	46	6	3.61 ± 2.23	45	6
Zhu (2018) [67]	Pig (Landrace × Large White)	Control	Normal	272	NA	C; P ^d^	85–114	0.126	P0	1390 ± 110	372	30	1340 ± 164	367	30
Proline															
Gonzalez-Anover (2017) [68]	Pig (Landrace × Yorkshire)	Control	Normal	115	14	C; P	11–30	0.120 ^e^	P0	1400 ± 755	844	57	1400 ± 762	835	58
**Aspartic acid**															
Brunner (1978) [43]	Rat (SD)	PKU-induction	FGR	0.22	NA	C; T	10–20	1.570 ^f^	20	2.60 ± 0.18	36	4	2.57 ± 0.36	36	4
		Control	Normal	0.22	NA	C; P	10–20	1.570 ^f^	20	2.96 ± 0.24	36	4	3.21 ± 0.24	36	4
**Valine**															
Brunner (1978) [43]	Rat (SD)	PKU-induction	FGR	0.22	NA	C; T	10–20	1.570 ^f^	20	2.52 ± 0.26	28	3	2.57 ± 0.36	36	4
								4.720 ^f^	20	2.42 ± 0.26	27	3	2.54 ± 0.31	108	12
		Control	Normal	0.22	NA	C; P	10–20	1.570 ^f^	20	2.86 ± 0.26	28	3	3.21 ± 0.24	36	4
								4.720 ^f^	20	3.15 ± 0.42	27	3	3.07 ± 0.21	108	12
Matsueda (1982) [97]	Rat (SD)	Control	Normal	0.19	6	C; P	1–22	2.710	22	3.49 ± 0.29	53	5	3.69 ± 0.21	53	5
Mori (1999) [98]	Rat (SD)	Control	Normal	0.19	20	I; P	12–22	2.368	22	5.20 ± 0.10	66	6	5.40 ± 0.40	99	9
Xu (2017) [104]	Pig (Landrace × Large White)	Control	Normal	220	18	C; P	107-P0	0.037	P0	1520 ± 396	78	8	1540 ± 396	82	8
								0.087	P0	1410 ± 396	80	8	1540 ± 396	82	8
**Leucine**															
Viana (2013) [103]	Mouse (NMRI)	Control	Normal	0.03	18	C; P ^d^	1–19	3.000 ^e,f^	19	0.95 ± 0.07	6	6	0.81 ± 0.03	6	6
Brunner (1978) [43]	Rat (SD)	PKU-induction	FGR	0.22	NA	C; T	10–20	1.570 ^f^	20	2.49 ± 0.30	36	4	2.57 ± 0.36	36	4
								4.720 ^f^	20	2.70 ± 0.21	27	3	2.54 ± 0.311	108	12
		Control	Normal	0.22	NA	C; P	10–20	1.570 ^f^	20	3.10 ± 0.24	36	4	3.21 ± 0.24	36	4
								4.720 ^f^	20	3.04 ± 0.21	27	3	3.07 ± 0.21	108	12
Cruz (2014) [99]	Rat (Wistar)	Control	Normal	0.25	18	C; P	10–20	NA	20	3.34 ± 0.24	24	8	3.67 ± 0.20	24	8
Cruz (2016) [100]	Rat (Wistar)	Control	Normal	0.25	18	C; P	2–20	NA	20	3.65 ± 0.19	101	10	3.92 ± 0.22	100	10
Matsueda (1982) [97]	Rat (SD)	Control	Normal	0.19	6	C; P	1–22	1.500	22	2.79 ± 0.31	49	5	2.05 ± 0.62	73	7
Mawatari (2004) [102]	Rat (SD)	Control	Normal	0.30	NA	I; P	7–17	0.300	20	4.11 ± 0.22	148 (M)	19	4.13 ± 0.29	128 (M)	19
										3.89 ± 0.27	136 (F)	19	3.89 ± 0.35	115 (F)	19
								1.000	20	4.12 ± 0.22	141 (M)	20	4.13 ± 0.29	128 (M)	19
										3.83 ± 0.17	151 (F)	20	3.89 ± 0.35	115 (F)	19
Mori (1999) [98]	Rat (SD)	Control	Normal	0.19	20	I; P	12–22	2.368	22	5.10 ± 0.40	45	5	5.40 ± 0.40	99	9
Ventrucci (2001) [101]	Rat (Wistar)	Control	Normal	0.15	18	C; P	1–20	7.180 ^f^	20	3.45 ± 0.51		10	3.73 ± 0.41		10
Wang (2018) [105]	Pig (Landrace × Large White)	Control	Normal	260	15	C; P	70-P0	0.046	P0	1470 ± 49	70	6	1490 ± 49	68	6
								0.092	P0	1550 ± 49	63	6	1490 ± 49	68	6
								0.138	P0	1460 ± 49	70	6	1490 ± 49	68	6
**Isoleucine**															
Brunner (1978) [43]	Rat (SD)	PKU-induction	FGR	0.22	NA	C; T	10–20	1.570 ^f^	20	2.53 ± 0.26	28	3	2.57 ± 0.36	36	4
								4.720 ^f^	20	2.88 ± 0.31	27	3	2.54 ± 0.31	108	12
		Control	Normal	0.22	NA	C; T	10–20	1.570 ^f^	20	3.24 ± 0.24	36	4	3.21 ± 0.24	36	4
								4.720 ^f^	20	2.95 ± 0.20	27	3	3.07 ± 0.21	108	12
Matsueda (1982) [97]	Rat (SD)	Control	Normal	0.19	6	C; P	1–22	3.150	22	3.81 ± 0.47	54	5	4.11 ± 0.22	47	5
Mori (1999) [98]	Rat (SD)	Control	Normal	0.19	20	I; P	12–22	2.368	22	5.30 ± 0.30	52	4	5.40 ± 0.40	99	9
Cysteine (NAC)															
Balansky (2009) [106]	Mouse (Swiss-Albino)	Control	Normal	0.03	NA	C; P	1-P0	1.000	P0	1.20 ± 0.04	47	5	1.30 ± 0.09	94	9
Moazzen (2014) [107]	Mouse (C57BL/6)	Streptozotocin	pre-existent DM	0.03	NA	C; P	0.5–18.5	1.000	P0	1.15 ± 0.07	28	8	0.99 ± 0.08	28	8
		Control	Normal	0.03	NA	C; P	0.5–18.5	1.000	P0	1.24 ± 0.08	28	8	1.24 ± 0.08	28	8
Miller (2014) [108]	Mouse (CD-1)	Control	Normal	NA	19	I; P	6–13	0.150	17	1.07 ± 0.38	353	25	1.00 ± 0.37	349	25
Lin (2011) [113]	Rat (SD)	HF diet	Overgrowth	0.23	17	C; P	1–19.5	0.075 ^e,f^	19.5	2.39 ± 0.19	213	20	2.38 ± 0.10	200	20
Soto-Blanco (2001) [114]	Rat (Wistar)	Control	Normal	0.27	16	C; P	6–21	0.510	21	4.37 ± 0.24	100	12	4.44 ± 0.24	87	12
Hashimoto (2012) [120]	Guinea pig (Dunkin-Hartley)	Hypoxia	FGR	NA	NA	C; T	52–62	0.550	63	59.8 ± 5.88		6	59.5 ± 5.9		6
		Normoxia	Normal	NA	NA	C; P	52–62	0.550	63	76.6 ± 19.1		6	88.8 ± 11.9		7
Herrera (2017) [121]	Guinea pig (Pirbright White)	Control	Normal	NA	19	C; P	34–67	0.500	67	79.1 ± 19.8	13		83.7 ± 12.4	16	NA
		UA occlusion	FGR	NA	19	C; P	34–67	0.500	67	74.1 ± 13.5	9		57.9 ± 15.3	9	
Thompson (2011) [122]	Guinea pig (Dunkin-Hartley)	Nicotine exposed	Risk	NA	NA	C; P	52–62	0.496	62	65.8 ± 5.4	NA	2	78.2 ± 6.4	NA	2
Motawei (2016) [133]	Human (Egypt)	PE	PE	85	NA	I; T	Mo 5-term (<4 months)	0.005	P0	2560 ± 590	50	50	198 ± 420	50	50
Roes (2006) [134]	Human (The Netherlands)	Early onset severe PE or HELLP	PE	72	NA	I; T	inclusion-delivery (±6 days)	0.025 ^e^	P0	9712 ± 419	19	19	1070 ± 399	19	19
Shahin (2009) [137]	Human (Egypt)	Previous preterm labor	Risk	80	NA	I; P	Wk 17–36 or P0	0.008 ^e^	P0	3107 ± 232	140	140	2715 ± 357	140	140
**Methionine**															
Abdel-Wanhab (1999) [115]	Rat (SD)	Control	Normal	0.20	NA	C; P	6–15	0.043	20	4.40 ± 0.33	69	8	4.50 ± 0.42	71	8
Brunner (1978) [43]	Rat (SD)	PKU-inducing diet	FGR	0.22	NA	C; T	10–20	1.570 ^f^	20	2.56 ± 0.18	36	4	2.57 ± 0.36	36	4
		Control	Normal	0.22	NA	C; P	10–20	1.570 ^f^	20	3.19 ± 0.24	36	4	3.21 ± 0.24	36	4
Chandrashekar (1977) [116]	Rat (SD)	Control	Normal	0.20	18	C; P	1–21	NA	21	2.30 ± 0.41	172	17	3.20 ± 0.59	368	35
Fujii (1971) [117]	Rat (McCollum)	Control	Normal	0.25	20	C; P	1-P0	0.029	P0	6.40 ± 0.16	NA (M)	4	6.40 ± 0.18	NA (M)	5
										6.10 ± 0.20	NA (F)	4	6.10 ± 0.13	NA (F)	5
Koz (2010) [118]	Rat (Wistar)	Control	Normal	0.25	NA	C; P	1-P0	1.000	P0	5.90 ± 0.30	25	5	6.10 ± 0.35	25	5
Matsueda (1982) [97]	Rat (SD)	Control	Normal	0.19	6	C; P	1–22	1.130	22	1.63 ± 0.27	45	5	2.05 ± 0.62	73	7
Mori (1999) [98]	Rat (SD)	Control	Normal	0.19	20	I; P	12–22	2.368	22	2.60 ± 0.10	88	8	5.40 ± 0.40	99	9
Viau (1973) [119]	Rat (SD)	Control	Normal	0.19	18	C; P	1–20	NA	20	2.50 ± 0.32	101	10	3.60 ± 0.28	82	8
Gauthier (2009) [123]	Guinea pig	Ethanol exposed	FGR	0.70	NA	C; P	35–71	0.096	71	99.0 ± 39.2	6	NA	98.0 ± 36.7	6	NA
Othmani Mecif (2017) [124]	Rabbit (White)	Control	Normal	2.90	30	C; P	0–29	0.172	29	36.0 ± 32.8	22	8	50.0 ± 26.8	45	8
Liu (2016) [125]	Sheep (Merino)	Control	Normal	63	NA	I; P	111-P0	0.047	P0	4620.0 ± 1929.6	79	60	4170.00 ± 1929.61	73	60
Tsiplakou (2017) [126]	Sheep (Chios)	Control	Normal	66	16	C; P	Last 15 days	0.041	P0	4.49 ± 0.66	16	15	4.51 ± 0.90	19	15
Batistel (2017) [128]	Cow (Holstein)	Control	Normal	735	16	C; P	Last 28 days	0.012 ^e^	P0	44,062 ± 5995	42	30	41,947 ± 5776	39	30
Clements (2017) [129]	Cow (Angus × Simmental)	Control	Normal	635	12	C; P	Last 23 days (±7 days)	0.016	P0	35,000 ± 4157	NA	6	35,000 ± 4156	NA	6
Jacometo (2016) [130]	Cow (Holstein)	Control	Normal	773	NA	C; P	Last 21 days	0.010 ^f^	P0	44,200 ± 5543	12	12	42,900 ± 5196	12	12
Xu (2018) [131]	Cow (Holstein)	Control	Normal	735	16	C; P	Last 28 days	0.012 ^e,f^	P0	41,760 ± 4432	NA	21	40,936 ± 6190	NA	18
**Choline**															
Bai (2012) [109]	Rat (Wistar)	Protein restriction	FGR	0.30	9	C; P	1-P0	0.670 ^e^	P0	5.72 ± 0.17	32 (M)	8	5.62 ± 0.20	32 (M)	8
										5.58 ± 0.17	32 (F)	8	5.35 ± 0.20	32 (F)	8
Thomas (2009) [110]	Rat (SD)	Ethanol exposed	FGR	0.20	NA	I; P	5–20	0.250	P0	6.90 ± 0.35	154	12	6.50 ± 0.33	160	11
		Control	Normal	0.20	NA	I; P	5–20	0.250	P0	6.90 ± 0.32	144	10	6.70 ± 0.37	200	14
Yang (2000) [111]	Rat (SD)	Control	Normal	NA	NA	C; P	11–17	0.390 ^e,f^	P0	6.35 ± 0.27	177	15	6.28 ± 0.24	155	12
Zhang (2018) [112]	Rat (SD)	LPS	FGR	0.20	NA	C; P	1–20	0.875 ^f^	20	4.02 ± 0.08	125	9	3.76 ± 0.13	105	9
		Control	Normal	0.20	NA	C; P	1–20	0.875 ^f^	20	4.35 ± 0.10	68	6	4.08 ± 0.06	79	6
Birch (2016) [127]	Sheep (Suffolk)	Ethanol infusion	FGR	75	13	C; P	4–148	0.010	P0	4940 ± 764	NA	8	4340 ± 1347	NA	14
		Control	Normal	75	13	C; P	4–148	0.010	P0	6150 ± 1592	NA	6	5740 ± 933	NA	8
Zenobi (2018a) [132]	Cow (Holstein)	Excess energy diet	Risk overgrowth	735	14	C; T	Last 21 days	0.024	P0	37,400 ± 7099	9	25	40,800 ± 7099	9	22
		Maintenance energy diet	Normal	735	14	C; P	Last 21 days	0.024	P0	39,300 ± 7099	9	21	40,100 ± 2099	8	25
Jacobson (2018) [138]	Human (South Africa)	Alcohol use	Risk	57	NA	I; T	Enrollment-P0	0.034	P0	2853 ± 451	31	31	2844 ± 658	31	31
Ross (2013) [135]	Human (USA)	Control	Normal	78	NA	I; P	Wk 17-P0	0.012 ^e^	P0	3114 ± 636	46	46	3193 ± 540	47	47
Yan (2012) [136]	Human (USA)	Control	Normal	63	NA	I; P	Wk 27–39	0.007 ^e^	P0	3500 ± 300	13	13	3400 ± 400	13	13

Ordered according to species form small to large per amino acid. ^a^ The supplementation scheme was continuous (C) or an interval (I). ^b^ Intervention type was treatment (T) or prevention (P). ^c^ When there is nothing in brackets reported in the table for sex of offspring (F or M) it means that the weight was measured in a mixed population. ^d^ Isonitrogenous control diet was used. ^e^ Dose in g/kg/day was calculated using the estimated mean maternal weight or based on ^f^ estimated food intake. “Normal” in the pregnancy complication column indicates the normal-growth group. AA, amino acid; BW, birth weight; DM, diabetes mellitus; EFW, estimated fetal weight; FGR, fetal growth restriction; GD, gestational day; L-NAME, (ω)-nitro-L-arginine methyl ester; LPS, lipopolysaccharides; NA: not applicable or available; HELLP, hemolysis, elevated liver enzyme, and low platelet syndrome; HTN, hypertension; P0, postnatal day 0 or day of P0; PE, preeclampsia; PKU; phenylketonuria; PIH, pregnancy-induced hypertension; RUPP, reduced uterine pressure perfusion; UA, uterine artery; UOL, unilateral oviduct ligation; WT, wild type; wk, weeks.

**Table A6:** Data extraction of included animal and human studies on maternal blood pressure.

Author (Year)	Species (Strain or Strain)	Model	Pregnancy Complication	Maternal Weight (kg)	Dietary Protein Intake (%)	Supplementation Scheme ^a^; intervention type ^b^	Daily Dose (g/kg Body wt)	Supplementation Duration (GD)	Technique (Office/Restrained vs. 24 h/Unrestrained)	Measurement Day (GD)	BP Type	AA BP ± SD (mmHg)	*n*	CON BP ± SD (mmHg)	*n*
**Arginine**															
Alexander (2004) [34]	Rat (SD)	RUPP	FGR/PE	0.25	NA	C; P	2.00 ^c^	10–19	Telemetry (unrestrained)	19	MAP	113 ± 6	9	132 ± 6	9
		Control	Normal	0.25	NA	C; P	2.00 ^c^	10–19	Telemetry (unrestrained)	19	MAP	97 ± 9	9	109 ± 7	12
Altun (2008) [35]	Rat (Wistar)	Sonic stress-induction	PE	0.20	NA	C; T	0.02	18–19	Tail cuff (restrained)	20	MAP	112 ± 10	6	145 ± 10	6
Bursztyn (2003) [45]	Rat (Wistar)	Hyper-insulinemia	FGR/PIH	0.24	NA	C; T	0.22	11-P0	Tail cuff (restrained)	19/20	SBP	77 ± 8	5	119 ± 14	5
Helmbrecht (1996) [48]	Rat (SD)	L-NAME	FGR/PE	0.31	NA	C; T	0.02	16–21	Tail cuff (restrained)	21	SBP	125 ± 7	9	153 ± 13	9
		Control	Normal	0.31	NA	C; P	0.02	16–21	Tail cuff (restrained)	21	SBP	117 ± 14	6	115 ± 7	6
Podjarny (1993) [90]	Rat (Wistar)	Control	Normal	0.23	20	C; T	0.32	12–22	Tail artery cannulation (restrained)	22	MAP	90 ± 7	10	91 ± 4	10
		Adriamycin nephropathy	PE	0.23	20	C; T	0.32	12–22	Tail artery cannulation (restrained)	22	MAP	90 ± 11	10	135 ± 13	10
Podjarny (2001a) [49]	Rat (Wistar)	Hyper-insulinemia	PIH	0.24	20	C; P	0.22	11–22	Tail cuff (restrained)	19/20	SBP	83 ± 9	15	104 ± 13	20
Podjarny (2001b) [91]	Rat (Wistar)	Control	Normal	0.23	20	C; P	0.26	11–22	Tail cuff (restrained)	22	SBP	87 ± 1	4	83 ± 6	4
		L-NAME	FGR/PE	0.23	20	C; T	0.26	11–22	Tail cuff (restrained)	22	SBP	89 ± 4	8	129 ± 4	8
Podjarny (1997) [92]	Rat (Wistar)	Adriamycin nephropathy	PE	0.22	20	C; T	0.23	11–22	Tail cuff (restrained)	22	SBP	106 ± 7	5	122 ± 7	8
											MAP	91 ± 5	5	124 ± 7	8
Schooley (2002) [50]	Rat (SD)	Mg deficiency	FGR	0.20	NA	C; T	1.00	10–21	Tail cuff (restrained)	21	SBP	111 ± 11	13	160 ± 10	12
		Control	Normal	0.20	NA	C; P	1.00	10–21	Tail cuff (restrained)	21	SBP	121 ± 11	13	148 ± 11	14
Sharkey (2001) [51]	Rat (SHHF)	Genetic	FGR/PIH	0.20	NA	C; P	0.10 ^c,d^	1–20	Tail cuff (restrained)	15–19	SBP	142 ± 14	3	188 ± 14	4
Camarena Pulido (2016) [78]	Human (Mexico)	Increased risk of PE	Risk	71	NA	I; P	0.04 ^c^	wk 20-P0	Sphygmo-manometer (office)	wk 39	SBP	118 ± 12	49	126 ± 14	47
											DBP	73 ± 11	49	79 ± 14	47
											MAP	88 ± 11	49	95 ± 5	47
Rytlewski (2005) [93]	Human (Poland)	PE; BW < p10	FGR/PE	75	NA	I; T	0.04	wk 29-P0	Sphygmo-manometer (office)	After 3 wks	SBP	134 ± 3	30	143 ± 3	31
											DBP	82 ± 2	30	87 ± 1	31
											MAP	102 ± 2	30	108 ± 1	31
Staff (2004) [89]	Human (Norway)	PE	PE	69	NA	I; T	0.18 ^c^	5 days	NA	After suppl.	SBP	147 ± 5	10	148 ± 5	8
											DBP	98 ± 3	10	102 ± 4	8
Neri (2010) [83]	Human (Italy)	Chronic HTN	Risk	90	NA	I; P	0.04 ^c^	10–12 wks	Sphygmo-manometer (24 h)	Aftersuppl.	SBP	129 ± 8	39	130 ± 14	40
									Sphygmo-manometer (24 h)	After suppl.	DBP	80 ± 7	39	79 ± 10	40
Winer (2009) [80]	Human (France)	AC < p3 and abnormal UA flow	FGR	75	NA	I; T	0.19	Until P0 (3.5 wks)	NA	After suppl.	SBP	133 ± 19	21	124 ± 18	22
											DBP	78 ± 13	21	74 ± 12	22
**Citrulline**															
Powers (2015) [94]	Human (USA)	Obese	Normal	85	NA	I; T	0.04 ^c^	16–19 wks	NA (office)	Wk 19	SBP	108 ± 4	12	116 ± 6	12
											DBP	66 ± 7	12	72 ± 6	12
**Cysteine**															
Motawei (2016) [133]	Human (Egypt)	PE	PE	85	NA	I; T	0.01	Mo 5-term (<4 mo)	NA (office)	After 6 wks suppl.	SBP	127 ± 8	50	133 ± 7	50
**Choline**															
Zhang (2018) [112]	Rat (SD)	LPS infusion	FGR	0.20	NA	C; P	0.88 ^d^	1–20	Tail cuff (restrained)	18	SBP	116 ± 2	9	130 ± 3	9
		Control	Normal	0.20	NA	C; P	0.88 ^d^	1–20	Tail cuff (restrained)	18	SBP	104 ± 2	6	103 ± 5	6
Jacobson (2018) [138]	Human (South Africa)	Alcohol	FGR	57	NA	I; T	0.03	wk 19-P0	Sphygmo-manometer (office)	12 wks after start	MAP	81 ± 9	34	85 ± 0	35

Ordered according to species per amino acid. ^a^ The supplementation scheme was continuous (C) or an interval (I). ^b^ Intervention type was treatment (T) or prevention (P). There were no studies using an isonitrogenous control diet. ^c^ Dose in g/kg/day was calculated using the estimated mean maternal weight or based on ^d^ estimated food intake. “Normal” in the pregnancy complication column indicates the normal-growth group. AC, abdominal circumference; BW, birth weight; DBP, diastolic blood pressure; GA, gestational age; FGR, fetal growth restriction; HTN, hypertension; IP, intraperitoneal; L-NAME, (ω)-nitro-L-arginine methyl ester; LPS, lipopolysaccharides; MAP, mean arterial pressure; Mg, magnesium; mo, months; NA, not applicable or available; P0, birth day; PE. preeclampsia; PIH, pregnancy-induced hypertension; RUPP, reduced uterine pressure perfusion; SBP, systolic blood pressure; SD, Sprague-Dawley; SHHF, spontaneous hypertension and heart failure; UA, umbilical artery; wks, weeks.

**Table A7:** Data extraction on development of pregnancy complication in risk population in human studies.

AA	Author (Year)	Country	Daily Dose (g/kgBW)	Pregnancy Complication	Definition	AA		CON	
						N Yes (%)	N No (%)	N Yes (%)	N No (%)
**Arginine**	Dera (2007) [95]	Poland	0.04 ^a^	SGA	BW < p10	24 (57)	18 (43)	15 (52)	13 (48)
	Camarena Pulido (2016) [78]	Mexico	0.04 ^a^	SGA	Undefined	1 (2)	48 (98)	3 (6)	44 (94)
				PE	≥140/90 mmHg ^b^	3 (6)	46 (94)	11 (23)	36 (77)
				Preterm	Undefined	1 (2)	48 (98)	7 (15)	40 (85)
				GDM	Undefined	2 (4)	47 (96)	2 (4)	45 (96)
	Facchinetti (2007) [79]	Italy	0.11 ^a^	SGA	BW < 2500 g	11 (28)	28 (72)	15 (43)	20 (57)
				PE	≥140/90 mmHg + proteinuria > 300 mg/24 h	3 (11)	24 (89)	7 (37)	12 (63)
				Preterm	GA < 37 wks	13 (16)	67 (84)	18 (25)	51 (74)
	Neri (2010) [83]	Italy	0.05 ^a^	SGA	BW < p10	7 (19)	32 (81)	10 (25)	30 (75)
				SGA	BW < 2500 g	7 (18)	32 (82)	11 (28)	29 (72)
				SGA	BW < 1500 g	1 (3)	38 (97)	5 (13)	35 (88)
				Preterm	GA < 37 wks	10 (26)	29 (74)	14 (35)	26 (65)
				Preterm	GA < 34 wks	2 (5)	37 (95)	7 (18)	33 (82)
	Rytlewski (2006) [85]	Poland	0.04	SGA	BW < p10	7 (23)	23 (77)	14 (45)	17 (55)
	Rytlewski (2008) [86]	Poland	0.04 ^a^	SGA	EFW < p10	8 (32)	17 (68)	8 (40)	12 (60)
	Sieroszewski (2004) [87]	Poland	0.04 ^a^	SGA	BW < p10	23 (29)	55 (71)	22 (75)	8 (25)
	Valdivia-silva (2009) [96]	Mexico	0.04	SGA	BW < p10	6 (6)	44 (94)	14 (30)	32 (70)
**Cysteine (NAC)**	Roes (2006) [134]	The Netherlands	0.03 ^a^	SGA	BW < p10	7 (37)	12 (63)	9 (47)	10 (52)
	Shahin (2009) [137]	Egypt	0.01 ^a^	Preterm	GA < 36 wks	8 (6)	132 (94)	62 (44)	78 (56)
				SGA	Undefined	4 (3)	136 (97)	12 (9)	128 (91)
**Choline**	Jacobson (2018) [138]	South Afrika	0.03 ^a^	SGA	BW < 2500 g	8 (25)	25 (75)	10 (32)	21 (68)
				PE	Undefined	2 (6)	32 (94)	3 (9)	32 (91)
				PIH	Undefined	3 (9)	31 (91)	4 (11)	31 (89)
				GDM	Undefined	4 (12)	30 (88)	0 (0)	35 (100)
	Ross (2013) [135]	USA	0.01 ^a^	SGA	Undefined	7 (15)	39 (85)	1 (2)	46 (98)
				PIH	Undefined	11 (24)	35 (76)	3 (6)	44 (94)
				Preterm	Undefined	9 (20)	37 (80)	7 (15)	40 (85)
				GDM	Undefined	3 (7)	43 (93)	2 (4)	45 (96)
				LGA	Undefined	2 (4)	43 (96)	1 (2)	45 (98)

Ordered according to amino acid. ^a^ Dose in g/kg/day was calculated using the estimated mean maternal weight. ^b^ According to American College of Obstetrics and Gynecology (ACOG). AA, amino acid; ACOG; BW, P0 weight; CON, control; EFW, estimated fetal weight; FGR, fetal growth restriction; GA, gestational age; GDM, gestational diabetes; LGA, large for gestational age; NAC, *N*-Acteyl Cysteine; PE, preeclampsia; PIH, pregnancy-induced hypertension; SGA, small for gestational age; wks, weeks.

**Table A8:** Data extraction included animal and human studies on gestational weight gain.

Author (Year)	Species (Strain or Country)	Animal Model	Pregnancy Complication	Mean Maternal Weight (kg)(kg)	Dietary Protein Intake (%)	Supplementation Scheme ^a^; Intervention Type ^b^	Supplementation Duration (GD)	Daily Dose (g/kg Body wt)	Measurement Day (GD)	AA Gestational Weight Gain (g)	*n*	CON Gestational Weight Gain (g)	*n*
**Arginine**													
Greene (2011) [33]	Mouse (FNVB/N × vegfr2-luc)	Control	Normal	0.03	24	C; T ^c^	1–18	2.660 ^d^	12–18	7 ± 1	6	6 ± 1	5
Bourdon (2016) [44]	Rat (SD)	Protein restriction	FGR	0.29	4	C; T ^c^	7–21	1.500	7–21	42 ± 17	9	42.5 ± 17	9
Bursztyn (2003) [45]	Rat (Wistar)	Hyper-insulinemia	FGR/PIH	0.24	NA	C; T	11-P0	0.217	0–22	88 ± 40	5	69 ± 35	5
Chin (1991) [46]	Rat (Fisher F344/NTacfBR)	Control	Normal	0.17	NA	C; P ^c^	1-P0	0.159	1-P0	18 ± 41	4	23 ± 41	4
Podjarny (1993) [90]	Rat (Wistar)	Control	Normal	0.23	20	C; T	12–22	0.322	0–22	151 ± 25	10	161 ± 43	10
		Adriamycin nephropathy	Risk	0.23	20	C; T	12–22	0.322	0–22	72 ± 23	10	76 ± 22	10
Podjarny (2001a) [49]	Rat (Wistar)	Hyper-insulinemia	PIH	0.24	20	C; P	11–22	0.220	0–22	78 ± 7	15	66 ± 8	20
Podjarny (2001b) [91]	Rat (Wistar)	Control	Normal	0.23	20	C; P	11–22	0.260	0–22	83 ± 11	4	86 ± 7	4
		L-NAME	FGR/PE	0.23	20	C; T	11–22	0.260	0–22	78 ± 15	8	64 ± 15	8
Vosatka (1998) [36]	Rat (Wistar)	Hypobaric hypoxia	FGR	0.25	4 g/day	C; P	9–21	2.000 ^d^	9–21	59 ± 10	7	39 ± 10	10
Zeng (2008) [37]	Rat (SD)	Control	Normal	0.23	22	C; P ^c^	0-P0	0.216	1–21	85 ± 22	12	79 ± 23	12
							1–7	0.216	1–21	75 ± 16	20	68 ± 17	20
Peine (2018) [53]	Sheep (Rambouillet-cross)	Diet restricted	FGR	68	16	C; P	54-P0	0.18	54–152	−7760 ± 2388	10	−7230 ± 2504	11
Sun (2017) [54]	Sheep (Hu)	Diet restricted twin bearing	FGR	40	NA	C; P	35–110	0.250	35–110	2600 ± 489	8	2600 ± 369	8
Sun (2018) [55]	Sheep (Hu)	Diet restricted twin bearing	FGR	40	10–14	C; T	35–110	0.249	1–110	4494 ± 3225	8	3983 ± 2518	8
Bérard (2010) [59]	Pig (Swiss Large White)	Control	Normal	159	NA	C; P	14–28	0.160	0–75	54,300 ± 9648	10	48,700 ± 9648	7
Bass (2017) [58]	Pig (GPK-35)	Control	Normal	180	19	C; P ^c^	93-P0	0.150	93–110	14,400 ± 6580	49	12,000 ± 6443	48
		Nulliparous	Normal	165	12	C; P ^c^	81–116	0.170	93–110	15,000 ± 6103	19	12,700 ± 64,416	21
		Primiparous	Normal	165	12	C; P ^c^	81–116	0.170	93–110	17,400 ± 5889	12	11,800 ± 6129	13
		Multiparous	Normal	165	12	C; P ^c^	81–116	0.170	93–110	11,500 ± 5940	18	12,700 ± 5613	14
Dallanora (2017) [69]	Pig (Landrace × Large white)	Control	Normal	148	17	C; P	25–112	0.115	80–112	22,900 ± 32,446	51	20,300 ± 32,446	51
Li (2010) [74]	Pig (Yorkshire × Landrace)	Control	Normal	113	12	C; P ^c^	0–25	0.070	0–25	4900 ± 15,973	9	7000 ± 15,973	9
							0–25	0.140	0–25	2700 ± 15,973	8	2700 ± 15,973	9
Li (2014) [75]	Pig (Yorkshire × Landrace)	Control	Normal	115	12	C; Pc	14–25	0.070	0–25	1100.0 ± 65,557	15	1100 ± 6557	14
							14–25	0.140	0–25	1200.0 ± 6557	14	1100 ± 6557	14
Li (2015) [76]	Pig (Landrace)	Control	Normal	125	16	C; P ^c^	1–30	0.21	1-P0	53,920 ± 1098	32	52,100 ± 10,980	30
				187	16	C; P ^c^	1–30	0.14	1-P0	47,800 ± 10,980	57	47,430 ± 10,980	56
				196	16	C; P ^c^	1–30	0.13	1-P0	46,380 ± 8964	37	45,640 ± 8964	37
Dera (2007) [95]	Human (Poland)	FGR < p10; gestational HTN	Risk	70	NA	I’T	Until P0	0.043 ^d^	Entry-P0	11,630 ± 4830	42	10,070 ± 3500	27
**Citrulline**													
Bourdon (2016) [44]	Rat (SD)	Protein restriction	FGR	0.29	4	C; T	7–21	2.000	7–21	54 ± 16	9	43 ± 17	9
Glutamate (NCG)													
Navarro (2005) [148]	Rat (Wistar)	Control	Normal	0.26	NA	C; P	2–20	0.110	2–20	116 ± 21	11	132 ± 16	7
Sun (2017) [54]	Sheep (Hu)	Diet restricted twin bearing	FGR	40	NA	C; P	35–110	0.125	35–110	4370 ± 433	8	2550 ± 369	8
Sun (2018) [55]	Sheep (Hu)	Diet restricted twin bearing	FGR	40	10–14	C; T	35–110	0.062	35–110	4200 ± 2605	8	2600 ± 369	8
Zhu (2015) [149]	Pig (Landrace × Yorkshire)	Control	Normal	132	NA	C; P	0–28	0.008	0–28	21,000 ± 7483	7	16,000 ± 10,198	8
Cai (2018) [66]	Swine (Landrace × Yorkshire)	Control	Normal	210	13	C; P	1–8	0.003	0–28	7810 ± 515	18	8390 ± 515	18
							9–28	0.003	0–28	8390 ± 515	16	8390 ± 515	18
							1–28	0.003	0–28	9350 ± 515	17	8390 ± 515	18
**Glutamine**													
Zhu (2018) [67]	Pig (Landrace × Large White)	Control	Normal	272	NA	C; Pc	85–114	0.126	84–110	40,700 ± 39,894	30	42,800 ± 31,773	30
**Aspartatic acid**													
Brunner (1978) [43]	Rat (SD)	PKU-induction	FGR	0.22	NA	C; T	10–20	1.570 ^e^	10–20	24 ± 22	4	30 ± 8	4
		Control	Normal	0.22	NA	C; P	10–20	1.570 ^e^	10–20	52 ± 18	4	44 ± 10	4
**Valine**													
Brunner (1978) [43]	Rat (SD)	PKU-induction	FGR	0.22	NA	C; T	10–20	1.570 ^e^	10–20	39 ± 11	3	30 ± 8	4
								4.720 ^e^	10–20	16 ± 4	3	30 ± 18	12
		Control	Normal	0.22	NA	C; P	10–20	1.570 ^e^	10–20	50 ± 17	3	44 ± 10	4
								4.720 ^e^	10–20	32 ± 2	3	55 ± 42	12
Matsueda (1982) [97]	Rat (SD)	Control	Normal	0.19	NA	C; P	1–22	2.71	1–22	40 ± 16	5	26 ± 12	5
Mori (1999) [98]	Rat (SD)	Control	Normal	0.19	20	I; P	12–22	2.368	12–22	95 ± 16	6	93 ± 34	9
**Leucine**													
Brunner (1978) [43]	Rat (SD)	PKU-induction	FGR	0.22	NA	C; T	10–20	1.570 ^e^	10–20	21 ± 22	4	30 ± 8	4
								4.720 ^e^	10–20	57 ± 6	3	29 ± 18	12
		Control	Normal	0.22	NA	C; P	10–20	1.570 ^e^	10–20	55 ± 28	4	44 ± 10	4
								4.720 ^e^	10–20	50 ± 17	3	55 ± 42	12
Matsueda (1982) [97]	Rat (SD)	Control	Normal	0.18	NA	C; P	1–22	1.500	1–22	−7.0 ± 11	5	−20 ± 17	7
Mawatari (2004) [102]	Rat (SD)	Control	Normal	0.30	NA	I; P	7–17	0.300	0–20	178 ± 31	19	158 ± 43	19
								1.000	0–20	179 ± 32	20	160 ± 43	19
Mori (1999) [98]	Rat (SD)	Control	Normal	0.19	20	I; P	12–22	2.368	12–22	82 ± 25	5	93 ± 34	9
Ventrucci (2001) [101]	Rat (Wistar)	Control	Normal	0.15	18	C; P	1–20	7.180 ^e^	1–20	61 ± 31	10	90 ± 36	10
**Isoleucine**													
Brunner (1978) [43]	Rat (SD)	PKU-induction	FGR	0.22	NA	C; T	10–20	1.570 ^e^	10–20	42 ± 19	3	30 ± 8	4
								4.720 ^e^	10–20	32 ± 4	3	29 ± 18	12
		Control	Normal	0.22	NA	C; P	10–20	1.570 ^e^	10–20	66 ± 28	4	44 ± 10	4
								4.720 ^e^	10–20	23 ± 19	3	55 ± 42	12
Matsueda (1982) [97]	Rat (SD)	Control	Normal	0.19	6	C; P	1–22	3.150	1–22	56 ± 11	5	44 ± 22	5
Mori (1999) [98]	Rat (SD)	Control	Normal	0.19	20	I; P	12–22	2.368	12–22	92 ± 9	4	93 ± 34	9
**Cysteine (NAC)**													
Miller (2014) [108]	Mouse (CD-1)	Control	Normal	0.03	19	I; P	6–13	0.150	6–17	14 ± 3	25	13 ± 3	25
Soto-Blanco (2001) [114]	Rat (Wistar)	Control	Normal	0.27	26	C; P	6–21	0.510	6–21	72 ± 16	12	68 ± 19	12
**Methionine**													
Brunner (1978) [43]	Rat (SD)	PKU-induction	FGR	0.22	NA	C; T	10–20	1.570 ^e^	10–20	23 ± 4	4	30 ± 8	4
		Control	Normal	0.22	NA	C; P	10–20	1.570 ^e^	10–20	44 ± 22	4	44 ± 10	4
Matsueda (1982) [97]	Rat (SD)	Control	Normal	0.19	NA	C; P	1–22	1.150	1–22	−54 ± 9	5	−20 ± 17	7
Mori (1999) [98]	Rat (SD)	Control	Normal	0.19	20	I; P	12–22	2.368	12–22	−16 ± 36	8	93 ± 34	9
Liu (2016) [125]	Sheep (Merino)	Control	Normal	63	NA	I; P	111-P0	0.047	111–1 wk pre-lambing	−1100 ± 1200	60	1500 ± 1200	60
**Choline**													
Bai (2012) [109]	Rat (Wistar)	Protein restriction	FGR	0.30	9	C; P	1-P0	0.670 ^d^	1–22	57 ± 7	8	59 ± 9	8
Zhang (2018) [112]	Rat (SD)	LPS infusion	FGR	0.20	NA	C; P	1–20	0.875 ^e^	1–20	175 ± 46	9	170 ± 24	9
Janovick Guretzky (2006) [151]	Cow (Holstein)	Control	Normal	715	NA	C; P	3 wks before P0	0.021	Last 2 wks	32,500 ± 49,041	5	5600 ± 58,138	5
	Cow (Jersey)	Control	Normal	506	NA	C; P	3 wks before P0	0.030	Last 2 wks	18,500 ± 47,518	16	12,800 ± 49,115	16
Jacobson (2018) [138]	Human (South Africa)	Alcohol use	Risk	57	NA	I; T	Enrollment-P0	0.035	NA	5960 ± 19,040	28	2020 ± 19,463	24
Ross (2013) [135]	Human (USA)	Control	Normal	78	NA	I; P	wk 17-P0	0.012 ^d^	NA	14,832 ± 8165	46	14,787 ± 6713	47
Yan (2012) [136]	Human (USA)	Control	Normal	63	NA	I; P	wk 27–39	0.007 ^d^	189–273	6000 ± 2200	13	6400 ± 2700	13

Ordered according to species per amino acid. ^a^ The supplementation scheme was continuous (C) or an interval (I). ^b^ Intervention type was treatment (T) or prevention (P). ^c^ Isonitrogenous control diet was used. ^d^ Dose in g/kg/day was calculated using the estimated mean maternal weight or based on ^e^ estimated food intake. “Normal” in the pregnancy complication column indicates the normal-growth group. BW, birth weight; FGR: fetal growth restriction; GD: gestational day; LPS, lipopolysaccharides; HTN, hypertension; PE: preeclampsia; PIH, pregnancy-induced hypertension; PKU; phenylketonuria; mo, month; NA: not applicable or available; P0, birth day; SD, Sprague Dawley; wk, wks.

**Table A9:** Data extraction of included animal and human studies on maternal glucose metabolism.

Author (Year)	Species (Strain)	Animal Model	Pregnancy Complication	Mean Maternal Weight (kg)(kg)	Dietary Protein Intake (%)	Supplementation Scheme ^a^; Intervention Type ^b^	Supplementation Duration (GD)	Daily Dose (g/kg Body wt)	Measurement Day (GD)	AA glucose ± SD (mmol/l)	*n*	CON Glucos ± SD (mmol/l)	*n*
**Arginine**													
Bursztyn (2003) [45]	Rat (Wistar)	Hyper-insulinemia	FGR/PIH	0.24	NA	C; T	11-P0	0.217	22	4.6 ± 1.6	5	4.4 ± 1.1	5
Podjarny (2001) [49]	Rat (Wistar)	Hyper-insulinemia	PIH	0.24	20	C; P	11–22	0.220	22	3.7 ± 0.6	15	4.1 ± 1.0	20
Zhang (2016b) [57]	Sheep (Hu)	Diet restricted twin bearing	FGR	40	NA	I; P	35–110	0.250	110	2.1 ± 1.0	8	1.7 ± 1.0	8
Li (2010) [74]	Pig (Yorkshire × Landrace)	Control	Normal	113	12	C; P ^c^	0–25	0.070	25	4.1 ± 0.8	9	4.1 ± 0.8	9
								0.140	25	4.0 ± 0.8	8	4.1 ± 0.8	9
Liu (2012) [77]	Pig (Landrace × Large White)	Control	Normal	187	15	C; P	1-P0	0.107		3.8 ± 0.7	9	3.5 ± 0.7	9
Glutamate (NCG)													
Zhang (2016) [57]	Sheep (Hu)	Diet restricted twin bearing	FGR	40	NA	I; P	35–110	0.060	110	2.2 ± 1.0	8	1.7 ± 1.0	8
Liu (2012) [77]	Pig (Landrace × Large White)	Control	Normal	187	15	I; P	1-P0	0.011	110	3.9 ± 0.7	9	3.5 ± 0.7	9
**Glutamine**													
Zhu (2018) [67]	Pig (Landrace × Large White)	Control	Normal	272	NA	C; P ^c^	85–114	0.126	100	4.0 ± 1.6	6	4.4 ± 1.6	6
									112	4.8 ± 1.6	6	4.7 ± 1.6	6
Leucine													
Ventrucci (2002) [150]	Rat (Wistar)	Control	Normal	0.15	18	C; P	1–20	7.180 ^e^	20	5.3	10	4.2 ± 0.7	10
Cysteine (NAC)													
Moazzen (2014) [107]	Mouse (C57BL/6)	Streptozotocin	Pre-existent DM	0.03	NA	C; P	0.5–18.5	1.000	18.5	23.9 ± 1.7	7	27.4 ± 5.3	7
		Control	Normal	0.03	NA	C; P	0.5–18.5	1.000	18.5	8.3 ± 0.5	7	7.6 ± 1.3	7
Lin (2011) [113]	Rat (SD)	High-fat diet	Over-growth	0.23	17	C; P	1–19.5	0.075 ^d,e^	19.5	6.2 ± 1.1	20	4.5 ± 0.8	20
**Choline**													
Zenobi (2018a) [132]	Cow (Holstein)	Excess energy diet	Risk overgrowth	735	14	C; T	Last 21 days	0.024	Mean of −12 and −7 prepartum	3.7 ± 0.6	25	3.6 ± 0.6	22
		Maintenance energy diet	Normal	735	14	C; P	Last 21 days	0.024	Mean of −12 and −7 prepartum	3.5 ± 0.6	21	3.6 ± 0.6	25
Zenobi (2018b) [152]	Cow (Holstein)	Diet restricted	FGR	732	10	C; P	Last 64 days	0.002	9	3.6 ± 0.7	15	3.5 ± 0.7	15
								0.003	10	3.5 ± 0.7	14	3.5 ± 0.7	15
								0.005	11	3.5 ± 0.7	14	3.5 ± 0.7	15
								0.007	12	3.3 ± 0.7	17	3.5 ± 0.7	15
		Ad libitum diet	Normal	732	15	C; P	Last 64 days	0.002	5	4.3 ± 0.5	15	4.2 ± 0.5	15
								0.003	6	4.2 ± 0.5	14	4.2 ± 0.5	15
								0.005	7	4.2 ± 0.5	14	4.2 ± 0.5	15
								0.007	8	4.1 ± 0.5	17	4.2 ± 0.5	15

Ordered according to species per amino acid. ^a^ The supplementation scheme was continuous (C) or an interval (I). ^b^ Intervention type was treatment (T) or prevention (P). ^c^ Isonitrogenous control diet was used. ^d^ Dose in g/kg/day was calculated using the estimated mean maternal weight or based on ^e^ estimated food intake. “Normal” in the pregnancy complication column indicates the normal-growth group. BW, birth weight; DM, diabetes mellites; FGR: fetal growth restriction; GD: gestational day; PE: preeclampsia; PIH, pregnancy-induced hypertension; mo, month; NA: not applicable or available; P0, birth day; SD, Sprague Dawley; wk, weeks.

**Table A10:** Quality assessment of included animal studies.

Author (Year)	Any Randomization	Any Blinding	Sample Size Calculation	Conflict of Interest statement	Free of Experimental Unit of Analysis Errors	Random Group Allocation (Selection)	Groups Similar at Baseline (Selection)	Blinded Group Allocation (Selection)	Random Housing (Performance)	Blinded Interventions (Performance)	Random Outcome Ass. (Detection)	Blinded Outcome Ass. (Detection)	Reporting of Drop-Outs (Attrition)	Selective Outcome Reporting (Reporting)	Other Biases
Abdel-Wanhab (1999) [115]	Y	N	N	N	N	?	?	?	?	?	?	?	L	?	?
Alexander (2004) [34]	N	N	N	N	Y	?	?	?	?	?	?	?	H	?	?
Altun (2008) [35]	N	N	N	N	Y	?	?	?	H	H	?	?	?	?	?
Bai (2012) [109]	Y	N	N	N	N	?	?	?	?	?	?	?	L	?	?
Balansky (2009) [106]	N	N	N	Y	N	?	?	?	?	?	?	?	L	?	L
Bass (2017) [58]	Y	N	N	N	Y	?	L	?	?	?	?	?	L	?	?
Batistel (2017) [128]	Y	N	N	Y	N	?	?	?	?	?	?	?	L	?	L
Beaudoin (1981) [40]	N	N	N	N	N	?	?	?	?	?	?	?	?	?	?
Bérard (2010) [59]	Y	N	N	N	Y	?	L	?	?	?	?	?	L	?	?
Birch (2016) [127]	Y	N	N	N	Y	?	?	?	?	?	?	?	?	?	?
Bourdon (2016) [44]	Y	N	N	Y	Y	?	L	?	?	?	?	?	H	?	L
Brunner (1978) [43]	N	N	N	N	N	?	?	?	?	?	?	?	?	?	?
Bursztyn (2003) [45]	N	N	N	N	N	?	L	?	?	?	?	?	L	?	?
Cai (2018) [66]	Y	N	N	Y	Y	?	L	?	?	?	?	?	H	?	L
Caporossi (2014) [42]	N	N	N	Y	N	?	?	?	?	?	?	?	?	?	L
Chandrashekar (1977) [116]	N	N	N	N	N	?	?	?	?	?	?	?	L	?	?
Che (2013) [70]	Y	N	N	N	Y	?	?	?	?	?	?	?	L	?	?
Chin (1991) [46]	Y	N	N	N	N	?	?	?	?	?	?	?	L	?	?
Clements (2017) [129]	Y	N	N	N	Y	?	?	?	L	?	?	?	L	?	?
Crane (2016) [52]	Y	N	N	N	Y	?	?	?	?	?	?	?	L	?	?
Cruz (2014) [99]	N	N	N	Y	N	?	?	?	?	?	?	?	?	?	L
Cruz (2016) [100]	N	N	N	Y	N	?	?	?	?	?	?	?	L	?	L
Da Costa (2014) [47]	Y	N	N	N	Y	?	?	?	H	?	?	?	L	?	?
Dallanora (2017) [69]	Y	N	N	Y	Y	?	?	?	?	?	?	?	L	?	L
Fuji (1971) [117]	N	N	N	N	N	?	?	?	?	?	?	?	?	?	?
Gao (2012) [71]	Y	N	N	N	Y	?	?	?	?	?	?	?	L	?	?
Garbossa (2015) [72]	N	N	N	N	Y	?	L	?	?	?	?	?	H	?	?
Gauthier (2009) [123]	Y	N	N	N	N	?	?	?	?	?	?	?	?	?	?
Gonzalez-Anover (2017) [68]	N	N	N	N	N	?	L	?	?	?	?	?	?	?	?
Greene (2011) [33]	Y	N	N	Y	Y	?	?	?	?	?	?	?	L	?	L
Guo (2016) [73]	Y	N	N	N	Y	?	?	?	?	?	?	?	L	?	?
Hashimoto (2012) [120]	N	N	N	Y	Y	?	?	?	?	?	?	?	H	?	L
Helmbrecht (1996) [48]	Y	Y	N	N	N	?	?	?	?	L	?	L	?	?	?
Herrera (2017) [121]	Y	N	N	Y	N	?	?	?	?	?	?	?	?	?	L
Jacometo (2016) [130]	Y	N	N	N	Y	?	?	?	?	?	?	?	H	?	?
Janovick Guretzky (2006) [151]	Y	N	N	N	Y	?	H	?	?	?	?	?	?	?	?
Koeners (2007) [38]	N	N	N	Y	N	?	?	?	?	?	?	?	?	?	L
Koz (2010) [118]	Y	N	N	N	N	?	?	?	?	?	?	?	L	?	?
Li (2010) [74]	Y	N	N	Y	Y	?	L	?	?	?	?	?	L	?	L
Li (2014) [75]	Y	N	N	Y	Y	?	L	?	?	?	?	?	L	?	L
Li (2015) [76]	Y	N	N	N	Y	?	L	?	?	?	?	?	L	?	?
Lin (2011) [113]	N	N	N	Y	Y	?	?	?	?	?	?	?	?	?	L
Liu (2012) [77]	Y	N	N	N	N	?	L	?	?	?	?	?	?	?	?
Liu (2016) [125]	Y	N	N	Y	N	?	?	?	?	?	?	?	?	?	L
Madsen (2017) [60]	Y	N	N	Y	Y	?	L	?	?	?	?	?	L	?	L
Mateo (2007) [61]	Y	N	N	N	Y	?	L	?	?	?	?	?	L	?	?
Mateo (2008) [62]	Y	N	N	N	Y	?	L	?	?	?	?	?	L	?	?
Matsueda (1982) [97]	N	N	N	N	N	?	L	?	?	?	?	?	H	?	?
Mawatari (2004) [102]	N	N	N	N	Y	L	L	?	?	?	?	?	H	?	?
Miller (2014) [108]	Y	N	N	N	Y	?	?	?	?	?	?	?	H	?	?
Moazzen (2014) [107]	N	N	N	Y	N	?	L	?	?	?	?	?	?	?	L
Mori (1999) [98]	N	N	N	N	N	?	?	?	?	?	?	?	?	?	?
Navarro (2005) [148]	N	N	N	N	N	?	N	?	?	?	?	?	?	?	?
Othmani Mecif (2017) [124]	N	N	N	Y	N	?	?	?	?	?	?	?	L	?	L
Peine (2018) [53]	Y	N	N	N	Y	?	L	?	?	?	?	?	L	?	?
Podjarny (2001a) [49]	N	N	N	N	N	?	L	?	?	?	?	?	?	?	?
Podjarny (2001b) [91]	N	N	N	N	Y	?	?	?	?	?	?	?	?	?	?
Podjarny (1993) [90]	N	N	N	N	N	?	N	?	?	?	?	?	?	?	?
Podjarny (1997) [92]	N	N	N	N	N	?	L	?	?	?	?	?	?	?	?
Quesnel (2014) [64]	N	N	N	N	N	?	L	?	?	?	?	?	H	?	?
Schooley (2002) [50]	N	N	N	N	N	?	L	?	?	?	?	?	H	?	?
Sharkey (2001) [51]	Y	N	N	N	N	?	L	?	?	?	?	?	L	?	?
Soto-Blanco (2001) [114]	N	N	N	N	Y	?	L	?	?	?	?	?	L	?	?
Sun (2018) [55]	Y	N	N	Y	N	?	?	?	?	?	?	?	L	?	L
Sun (2017) [54]	Y	N	N	Y	Y	?	?	?	?	?	?	?	L	?	L
Thomas (2009) [110]	Y	N	N	Y	Y	?	?	?	?	?	?	?	?	?	L
Thompson (2011) [122]	N	N	N	Y	N	?	?	?	?	?	?	?	?	?	L
Tran (2017) [39]	Y	N	N	Y	N	?	?	?	?	?	?	?	?	?	L
Tsiplakou (2017) [126]	N	N	N	N	N	?	?	?	?	?	?	?	?	?	?
Ventrucci (2001) [101]	N	N	N	N	N	?	L	?	?	?	?	?	L	?	?
Ventrucci (2002) [150]	N	N	N	Y	N	?	L	?	?	?	?	?	L	?	L
Viana (2013) [103]	Y	N	N	N	Y	?	?	?	?	?	L	?	L	?	?
Viau (1973) [119]	N	N	N	N	Y	?	?	?	?	?	?	?	L	?	?
Vosatka (1998) [36]	Y	N	N	N	N	?	?	?	?	?	?	?	H	?	?
Wang (2018) [105]	Y	N	N	Y	Y	?	?	?	?	?	?	?	H	?	L
Wu (2012) [63]	Y	N	N	N	Y	?	L	?	?	?	?	?	L	?	?
Xu (2017) [104]	N	N	N	N	Y	?	L	?	?	?	?	?	L	?	?
Xu (2018) [131]	N	N	N	N	N	?	?	?	?	?	?	?	H	?	?
Yang (2000) [111]	Y	Y	N	N	N	?	?	?	?	?	?	?	L	?	?
Zeng (2008) [37]	Y	N	N	Y	Y	?	L	?	?	?	?	?	L	?	L
Zeng (2012) [41]	Y	N	N	Y	N	?	?	?	?	?	?	?	L	?	L
Zenobi (2018a) [132]	Y	Y	N	N	Y	?	?	?	?	?	?	?	H	?	?
Zenobi (2018b) [152]	Y	N	N	N	N	?	?	?	?	?	?	?	L	?	?
Zhang (2014) [65]	Y	N	N	Y	Y	?	L	?	?	?	?	?	L	?	L
Zhang (2016a) [56]	Y	N	N	Y	Y	?	L	?	?	?	?	?	L	?	L
Zhang (2016b) [57]	Y	N	N	Y	N	?	L	?	?	?	?	?	L	?	L
Zhang (2018) [112]	Y	N	N	Y	N	?	?	?	?	?	?	?	L	?	L
Zhu (2018) [67]	Y	N	N	Y	Y	?	L	?	?	?	L	?	L	?	L
Zhu (2015) [149]	Y	N	N	Y	Y	?	L	?	?	?	?	?	L	?	L

Quality assessment using the SYRCLE risk of bias tool: The first five columns represent the reporting of key study quality indicators; the last ten columns entail the risk of bias assessment. Y = yes, reported; N = no, not reported; H = High risk of bias; L = Low risk of bias; ? = unclear risk of bias.
